# Two-dimensional photonic MXene nanomedicine

**DOI:** 10.1515/nanoph-2022-0514

**Published:** 2022-10-24

**Authors:** Ruxi Deng, Meiqi Chang, Yu Chen, Yang Zhou

**Affiliations:** Department of Ultrasound, The Third People’s Hospital of Chengdu, The Affiliated Hospital of Southwest Jiaotong University, Chengdu 610031, P. R. China; Materdicine Lab, School of Life Sciences, Shanghai University, Shanghai 200444, P. R. China; Central Laboratory of Shanghai Municipal Hospital of Traditional Chinese Medicine, Shanghai University of Traditional Chinese Medicine, Shanghai 200071, P. R. China

**Keywords:** 2D MXenes, photodynamic therapy, photonic antibacteria, photonic bioimaging, photothermal therapy

## Abstract

Two-dimensional (2D) transition metal carbides and nitrides (MXenes) with fascinating physicochemical properties, ultrathin lamellar structure, high specific surface area, and excellent biocompatibility have been extensively explored in biomedical applications over the past decade. Photonic MXene nanomedicine and materdicine, as one of the most burgeoning emerging treatment modalities, are of great research interest owing to their photon utilization ability and high therapeutic efficiency. This review aims to summarize and discuss the very-recent advances in engineering 2D MXenes for photonic theranostic applications. We initially concentrate on the synthesis methods, surface modification, and functionalization with respect to MXenes. Furthermore, the photonic biological applications including photonic antibacteria, photonic bioimaging, photonic therapy, and photonic theranostics are highlighted in detail with the selected paradigms. Finally, the current challenges and future directions for 2D MXene biomaterials in regard to their photonic biomedicines are discussed in depth, aiming to drive the speed of their practical applications in clinic.

## 1 Introduction

In recent years, two-dimensional (2D) materials featuring specific sheet-like nanostructure and morphology, which are distinctive from their bulk counterparts and nanoparticles, have attracted great interests because of their exceptional physicochemical properties, such as quantum size effect and large surface area [1]. The advent of graphene has promoted the development of the synthesis and application of a plethora of 2D materials. Since then, two-dimensional 2D materials consisting of black phosphorus (BP) [2, 3], hexagonal boron nitride (*h*-BN) [4], transition metal dichalcogenides (TMDs) [5], 2D metal-organic frameworks (MOFs) [6], layered double hydroxides (LDHs) [7, 8], graphitic carbon nitride nanosheets (g-C_3_N_4_) [9], monoelemental materials (Xenes: *e.g.*, gallenene (2D gallium), silicene (2D silicon), stanine (2D tin), phosphorene (2D phosphorus), antimonene (2D antimony), bismuthene (2D bismuth), tellurene (2D tellurium), iodinene (2D iodine)) [10–12], covalent organic frameworks (COFs) [13], metallene [14], *etc*., have been extensively explored. In the available researches, the library of 2D biomaterials has been extensively used in the field of biomedicine, including drug delivery [15], diagnostic imaging [16], disease therapy [17], *etc*. (Table 1).

**Table 1:** Summary of photonic biomedical applications of 2D MXenes.

Materials	Photonic biomedical applications	Ref.
Ti_3_C_2_T_*x*_	Antibacterial	[18]
Bi_2_S_3_/Ti_3_C_2_T_*x*_	Antibacterial	[19]
Ti_3_C_2_@Au-PEG	Antibacterial	[20]
Ti_3_C_2_T_*x*_–Au	Antibacterial	[21]
Nb_2_C	Antibacterial	[22]
V_2_C	Antibacterial	[23]
MnO_*x*_/Ta_4_C_3_	PA imaging	[24]
Ti_3_C_2_@Au	PA imaging	[25]
Ti_2_N QDs	PA imaging	[26]
Ti_3_C_2_ QDs	Fluorescence imaging	[27–29]
Nb_2_C QDs	Fluorescence imaging	[30, 31]
Ta_4_C_3_-SP	Multiple bio-imaging	[32]
W_1.33_C-BSA	Multiple bio-imaging	[33]
V_2_C-TAT@Ex-RGD	Multiple bio-imaging	[34]
MnO_*x*_/Ta_4_C_3_	Multiple bio-imaging	[24]
MnO_*x*_/Ti_3_C_2_	Multiple bio-imaging	[25]
Ti_3_C_2_-SP	PTT	[35]
MnO_*x*_/Ti_3_C_2_	PTT	[25]
MnO_*x*_/Ta_4_C_3_	PTT	[24]
Ta_4_C_3_-IONP-SPs	PTT	[36]
Nb_2_C	PTT	[37, 38]
Mo_2_C	PTT	[39]
V_2_C	PTT	[40]
Ti_3_C_2_ QDs	PTT	[41]
V_2_C-TAT@Ex-RGD	PTT	[34]
Ti_2_C-PEG	Synergistic photonic therapy	[42]
Mo_2_C	Synergistic photonic therapy	[43]
Ti_3_C_2_@DOX@HA	Synergistic photonic therapy	[44]
Ti_3_C_2_@Met@CP	Synergistic photonic therapy	[45]
Ti_3_C_2_@IR780	Synergistic photonic therapy	[46]
Ti_3_C_2_@Au	Photonic theranostics	[47]
Fe(II)-Ti_3_C_2_	Photonic theranostics	[48]
Ta_4_C_3_-IONP-SPs	Photonic theranostics	[36]
MnO_*x*_/Ta_4_C_3_	Photonic theranostics	[24]
V_2_C	Photonic theranostics	[49]
V_2_C-TAT@Ex-RGD	Photonic theranostics	[34]

Among these 2D materials, MXenes are the emerging multifunctional inorganic compounds consisting of transition metal carbides, carbonitrides, and nitrides [50, 51], which share a chemical formula of *M*_*n*+1_ *X*_*n*_ *T*_*x*_ (*n* = 1–6), where *M* represents an early transition metal (Sc, Ti, Zr, Hf, V, Nb, Ta, Cr, Mo), *X* denotes a carbon or nitrogen and *T*_*x*_ represents different surface terminations (O, OH, F, Cl, Br, S, Se, Te) [52]. The presence of plentiful surface functional groups (hydroxyl, terminal oxygen, and fluorine) endows MXenes with hydrophilic nature and the derived exciting electronic, optical, and magnetic properties. Compared to other 2D materials, MXene has a wide variety of surface compositions allowing tuning of band gap and surface plasmon resonance effects, which are necessary for nanodynamic therapy [53]. In addition, the transition metal elements in MXenes have catalytic activity for nanocatalytic therapy. MXenes also have desirable biodegradability and biocompatibility, facilitating their biomedical applications. Since the initial discovery of Ti_3_C_2_ in 2011 [54], there has been rapid progress in versatile biomedical applications of 2D MXene nanomaterials. Distinct from other 2D materials, mono- or few-layer MXene nanosheets could achieve rapid biodegradation due to their environmental (oxygen and water) instability [55], substantially enhancing their clinical application value and potential.

The distinctive optical properties including light absorption, emission and scattering accelerate the photonic biomedical applications of 2D MXene materials. When light activates perpendicularly on a surface, changes in photon energy may be an indication of interactions between photons and MXenes in the plane. This is because light is an electromagnetic wave whose electric and magnetic fields are perpendicular to each other and perpendicular to the direction of the energy and waves [56]. Particularly, the photonic-harvesting property of MXenes has attracted dramatically impressive attention. The broadband absorption characteristic in the near-infrared (NIR) region including both the first and second NIR biological windows endowed MXene with the excellent ability to be efficiently used in biomedicine. It has been demonstrated that MXenes feature a high photothermal conversion efficiency, which in some circumstances could approach 50% [37]. The photonic features might be altered through the synthesis of distinct MXenes with biological applications.

**Figure 1:**
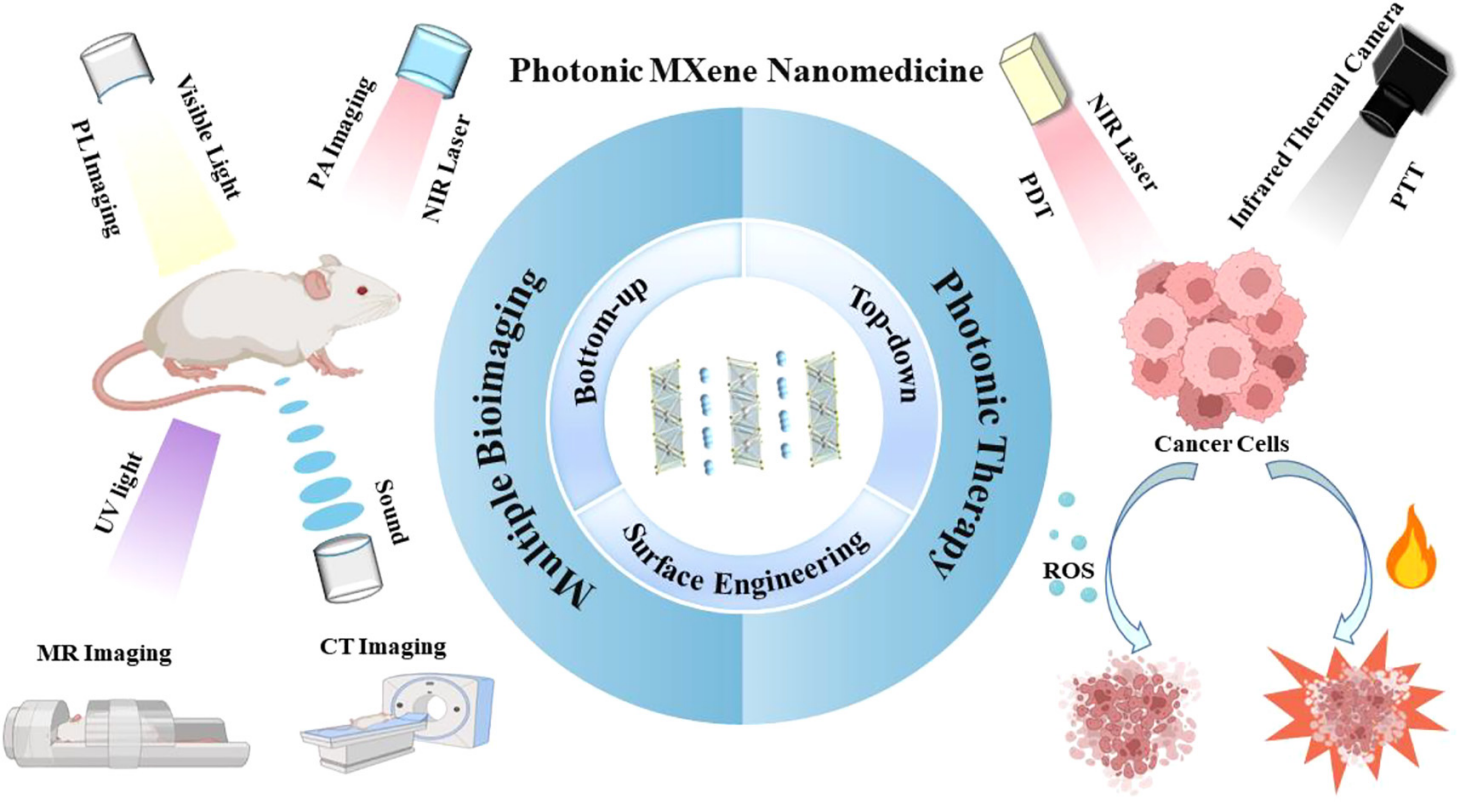
A review of 2D photonic MXene used in nanomedicine. Biological applications based on 2D photonic MXene, including bioimaging and therapy.

The state-of-the-art developments of MXene-based biomaterials in photonic biomedical applications have been thoroughly reviewed and carefully summarized in this comprehensive review. The introduction of MXene fabrication techniques, as well as their surface alterations, comes first. Then, we clarify how 2D MXenes can be engineered for a variety of photonic biomedical applications, including photonic antimicrobial, photonic imaging, photonic therapy, and photonic theranostics (Figure 1). Finally, we elaborate on the prospects and obstacles for further development of 2D MXene-based nanomedicine and biomaterials, with the goal of accelerating the advancement of photonic biomedical applications in clinic.

## 2 Synthesis and properties and 2D MXenes

**Figure 2:**
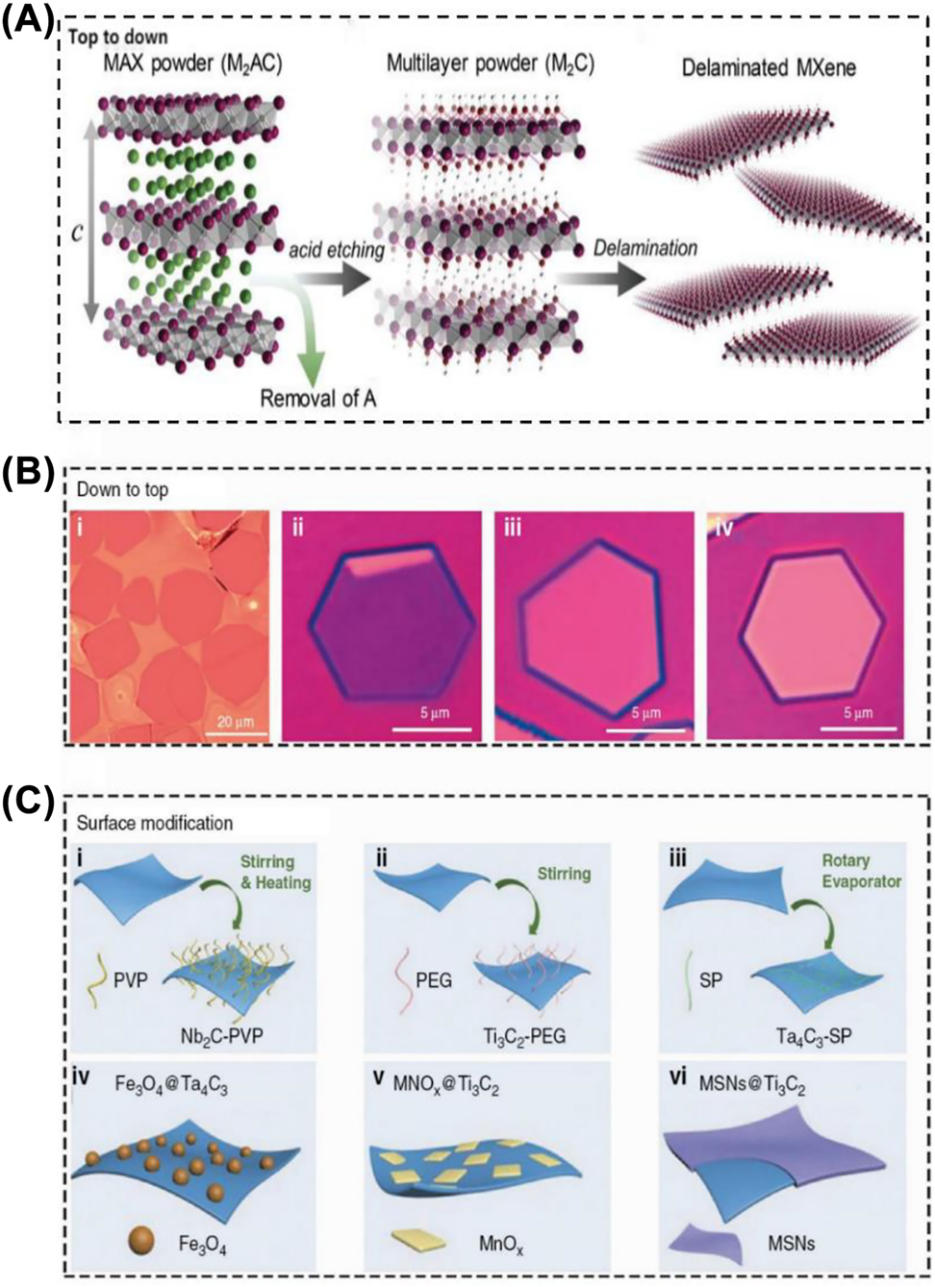
Synthesis and modification of MXene. (A) Schematic diagram of the top-down approach for MXene nanosheets [57]. Copyright 2019, Wiley-VCH. (B) i, optical images of Mo_2_C crystal fabricated by the bottom-up approach; ii–iv, optical images of Mo_2_C crystal on silica/Si substances of different thicknesses (6.7, 8.2, and 11.2 nm). (C) Schematic diagram of MXene surface modification: i, Nb_2_C nanosheet surface modified with PVP; ii, Ti_3_C_2_ nanosheet surface modified with PEG; iii, Ta_4_C_3_ nanosheet surface modified with SP; iv, Ta_4_C_3_ surface modified with Fe_3_O_4_; v, Ti_3_C_2_ nanosheet surface modified with MnOx; vi, Ti_3_C_2_ nanosheet modified with MSNs [17]. Copyright 2020, De Gruyter.

To date, more than 40 MXenes have been experimentally synthesized. The reported preparation methodologies can be generally divided into two categories: top-down methods (*i.e.*, selective etching, delamination strategies) and bottom-up fabrication (Figure 2A and B) [17, 57]. In addition, various surface modification strategies have been exploited to enhance their versatility and biomedical properties (Figure 2C) [17].

### 2.1 Top-down methods

The principal exfoliation method is the typical top-down preparation of MXenes due to the similar layered structure between the bulk precursors of MXenes and other 2D materials. In general, there have been two main steps for the acquisition of 2D MXenes from bulk MAX phases: (i) selective etching using appropriate etchants to obtain multilayered MXenes, and (ii) delamination using organic molecules/cations insertion, tilting, and/or ultrasonic treatment to acquire single- or few-layer MXenes. Considering MXenes synthesized by top-down methods are not suitable for biomedical applications due to the inappropriate size of initial products, the lateral dimensions and thickness of MXenes materials need to be further optimized by various methods, such as filtration [58], differential centrifugation [59], ultrasonication [60], *etc*.

#### 2.1.1 Selective etching strategies

The selective etching strategies, including direct hydrofluoric acid (HF) etching, HF-forming etching, HF-containing etching, molten-slats etching, and alkali etching, play a critical role in acquiring single- or few-layer MXenes from MAX phases. Starting from the etching of the Ti_3_AlC_2_ MAX phase to obtain Ti_3_C_2_ MXenes [54], HF etching is widely used as a convenient and effective method for the etching MAX phase bulks. It is well-known that the preparation of a large number of MXenes requires the participation of the HF etching process, such as Ti_2_CT_*x*_, Ta_4_C_3_T_*x*_, Nb_2_CT_*x*_, V_2_CT_*x*_, Nb_4_C_3_T_*x*_, Mo_2_CT_*x*_, Zr_3_C_2_T_*x*_, Hf_3_C_2_T_*x*_ [61], *etc*. HF etching not only etches the MAX phase effectively and selectively but also endows MXenes materials with an accordion-like morphology. Specifically, different MAX phases need the distinct etching conditions. For example, it takes more than three days to obtain Nb_2_C MXenes by immersing the Nb_2_AlC MAX phase into a 50 wt% HF solution at room temperature [62], whereas the complete etching of Ti_3_AlC_2_ MAX phase needs only about one day in a 5 wt% HF solution [63]. In addition, the etching conditions are related to the strength of the A-bond. In general, it is easier to obtain MXenes from MAX phases that are attached to relatively weak ionic bonds *via* Al, Si, or Ga [64]. Furthermore, diverse HF concentrations can yield MXenes with different surface terminations (*e.g.*, –O, –OH, and/or –F) and distinguishable defects density. A lower concentration of HF solution generally results in the acquisition of MXenes with more –O and less –F terminations, whereas a higher concentration of HF solution provides MXenes with stronger interlayer interactions and more defects. Besides, both mono-metal or double-metal MXenes (*e.g.* Cr_2_TiC_2_T_*x*_, Mo_2_Ti_2_C_3_T_*x*_, and Mo_2_TiC_2_T_*x*_) have been successfully synthesized in a varied concentrations of HF solution by etching their MAX phase bulks [65]. It can be concluded that selection of suitable etching conditions signifies the pivotal role of obtaining MXenes materials with high yield and purity features.

Considering that a trace amount of HF is harmful to biological organisms, a series of alternative approaches have been exploited and developed to avoid HF-related security issues. For instance, in 2014, Ghidiu et al. figured out a milder selective etching strategy to fabricate MXenes by mixing Ti_3_AlC_2_ powders, fluoride salts (*e.g.*, NaF, KF, LiF, and CsF) with HCl or H_2_SO_4_ [66]. Subsequently, Lipatov et al. improved the selectively etching method by changing the molar ratio of LiF to MAX and the concentration of HCl, aiming to produce high-quality monolayer MXenes with high O/F ratio, few defects, low resistivity, and excellent environmental stability. It is worth mentioning that the synthetic MXenes materials could remain stable after being exposed to air for even 70 h [67]. Intriguingly, Halim et al. successfully prepared MXenes with hydrogen ammonium bifluoride ((NH_4_HF_2_) instead of LiF [68], further broadening the variety of fluorine-based etchants.

Despite different strategies had been developed and proposed to avoid or reduce the utilization of HF, there are still inevitable problems regarding safety and environmental pollution. In view of this situation, Li et al. demonstrated that a fluorine-free strategy, namely the alkali etching strategy, is capable of obtaining MXenes from MAX phase bulks [69]. In this procedure, the typical Ti_3_C_2_T_*x*_ (T = –OH, –O) was synthesized at 270 °C in a 27.5 M NaOH solution with 92 wt% purity [69]. The temperature and NaOH concentration are two important factors in this reaction, in which the temperature control determines the successful production of Ti_3_C_2_T_*x*_, while the NaOH concentration is closely related to the purity of the product. In addition, Li et al. prepared various MXenes through etching MAX-phase precursors with a elements (Si, Zn, and Ga) by Lewis acidic molten salts (*e.g.*, CuCl_2_, NiCl_2_, AgCl, FeCl_2_, CdCl_2,_ and CoCl_2_) [70]. This generic selective etching strategy based on Lewis acidic molten salts enlarges the range of MAX phases and the potential properties of MXenes *via* tailoring the surface chemistry.

#### 2.1.2 Delamination strategies

Weakening the interaction force between the adjacent layers plays a crucial role of the delamination of MXenes since multilayer MXenes synthesized by selective etching are held together by van der Waals interactions and/or hydrogen bonding. It is well-known that intercalating large organic molecules or ions, shanking, and/or sonication are effective approaches to obtain single- or few-layer MXenes [71]. Moreover, the delamination route depends on etching conditions. Spontaneous intercalation of cations *(e.g.,* Li^+^, Na^+^, K^+^, Mg^2+^, and NH_4_^+^) was used to intercalate into the multilayered MXenes because the van der Waals force can be weakened by the subsequent negative surface charges of MXenes [72]. Apart from this, Ghidiu et al. made the Li^+^ ions intercalate between the adjacent MXenes sheets through the HCl/LiF approach and utilized simple shaking or sonication to facilitate the exfoliation process [66]. It is vital to note that the successful delamination of MXenes *via* HF etching strategy can only intercalate ions or organic molecules among the layers. Otherwise, the chemical compositions are related to the delamination strategy as well. For instance, when the exfoliation process of Ti_3_C_2_ [73] and (Mo_2/3_Ti_1/3_)_3_C_2_ [65] MXenes is insensitive for etching reagent with different compositions, it was reported that dimethyl sulfoxide could be employed instead.

### 2.2 Bottom-up synthesis

In terms of bottom-up fabrication techniques, they often start with tiny organic or inorganic molecules or atoms that have been organized into a 2D-ordered structure by crystal formation, whereas top-down production techniques start with bulk precursors [64]. It is worth mentioning that the bottom-up synthesis strategy enables researchers to accurately control the size distribution, morphology, compositions, and surface termination of MXenes *via* regulating the experimental procedure. However, very few bottom-up methods have been reported, presumably due to the complex elementary composition and crystal lattice. In 2015, the classic synthesis strategy-chemical vapor deposition (CVD) was used to fabricate MXenes [74]. In this procedure, the high-quality ultrathin Mo_2_C crystals were acquired by using a Cu/Mo foil under a methane atmosphere above 1085 °C. These crystals are extremely stable in their surroundings and are over 100 µm in size. However, due to their defect-free, high crystallinity, and surface termination group flaws, films made using this approach are undesirable for surface engineering and modification in biomedical applications. In addition, the same procedure can be used to create ultrathin WC and TaC crystals from additional transition metals, including W and Ta. Except for CVD method, MXenes can also be fabricated by plasma-enhanced pulsed laser deposition (PELPD) and template method [75, 76]. In 2017, Zhang et al. synthesized ultrathin Mo_2_C with the face-centered cubic structure using Mo vapor produced by pulsed laser and methane plasma as the carbon source [75].

### 2.3 Surface modification

MXenes are generally hydrophilic based on the specific surface terminations (such as –OH, –O, and –F), which contributes to their high stability in colloidal aqueous solutions without surfactants [77, 78]. The delaminated MXenes nanosheets, however, typically exhibit considerable aggregation and precipitation and lack numerous functions under physiological settings [79, 80]. Therefore, the suitable surface functionalization/modification plays a crucial role in improving their biomedical performance, such as their biocompatibility, dispersity, circulation, stability, loading capacity, and targeting ability [81]. Generally, surface modification methods are broadly categorized into non-covalent and covalent modifications.

#### 2.3.1 Non-covalent strategies

In general, MXenes that have been synthesized in the past typically perform poorly in terms of stability and dispersibility in physiological solutions. MXenes must undergo the proper surface modification, such as non-covalent interaction, in order to be feasibly used in biomedicine. Some natural molecules, for instance, soybean phospholipid (SP) with the features of sufficient surface area and favorable biodegradability could modify MXenes by means of physical absorption. According to these characteristics, our team created a Ti_3_C_2_-SP nanosystem for intravenous injection that has sufficient stability, permeability, and biocompatibility in order to increase the effectiveness of tumor-thermal therapy [35]. Electrostatic attraction, in addition to physical absorption, is a successful tactic for non-covalent interactions, because the negative charge on MXenes’ surface makes it simple for positively charged molecules to adhere. Both polyethylene glycol (PEG)-modified Ti_3_C_2_ MXene [82] and polyvinylpyrrolidone (PVP)-modified Nb_2_C MXene [37] exhibited exceptional stability under different physiological conditions. Moreover, it turns out that polyethyleneimine (PEI) [83] and poly (dopamine) (PDA) [84] also show satisfactory results in inhibiting the aggregation and deposition of MXenes nanosheets.

#### 2.3.2 Covalent modifications

In addition to non-covalent methods, covalent modification is a prominent way to improve the biocompatibility of MXenes. In the past few years, self-initiated photographing-photopolymerization (SIPGP) has been demonstrated to effectively modify the surface of miscellaneous materials (*e.g.*, graphene, diamond, carbon nanotubes, and silicon-based materials) [85–87]. Intriguingly, SIPGP can be branched straightly to the surface of materials under ultraviolet (UV) irradiation at room temperature, while other polymerization methods require catalysts, ligands, and other reaction conditions. In 2015, Chen et al. synthesized MXenes with dual response properties of CO_2_ and temperature *via* grafting poly (2-(dimethylamino) ethyl methacrylate) (PDMAEMA) on the surface of V_2_C MXene through SIPGP, opening the door for their use in biological applications [88]. Additionally, there seems to be a consensus on the ability of aryl diazonium to construct covalent bonds on the surface of various materials such as metal nanoparticles, metal oxides, carbon materials, and semiconductors [89, 90]. Wang et al. utilized the aryl diazonium salt to modify the surface of as-prepared Ti_3_C_2_ MXene nanosheets by forming powerful Ti–O–C bonds [91]. These nanosheets are improved in terms of dispersion, stability, and specific surface area through this method. Moreover, this modification method allows for the covalent modification of additional functional molecules on the surface of MXenes by employing aryl diazo ions as surface modifiers and inserters, thus opening up a wide range of potential applications for 2D materials. However, to our knowledge, the use of aryl diazo to functionalize the surfaces of MXene-related biomaterials is still in its early stages of development.

### 2.4 Photonic properties

MXenes are a distinct class of multifunctional 2D nanomaterials with unique electronic, magnetic, thermal, and mechanical properties that set them apart from other types of materials [92]. Generally, the optical properties of MXenes including light absorption, scattering, and emission are believed to hold the key to biomedical applications. VahidMohammadi et al. summarized that the optical properties of MXenes are connected to the structure and type of the M and X sites and the stoichiometry of surface termination groups [93]. For instance, M_2_C (M = Ti, Hf, Zr) MXenes with appropriate band gaps (0.92–1.75 eV) are efficient visible-light-driven photocatalysts [94]. Additionally, Magne et al. modified the surface terminations of MXenes to adapt the band gap to fit the UV light [95].

**Figure 3:**
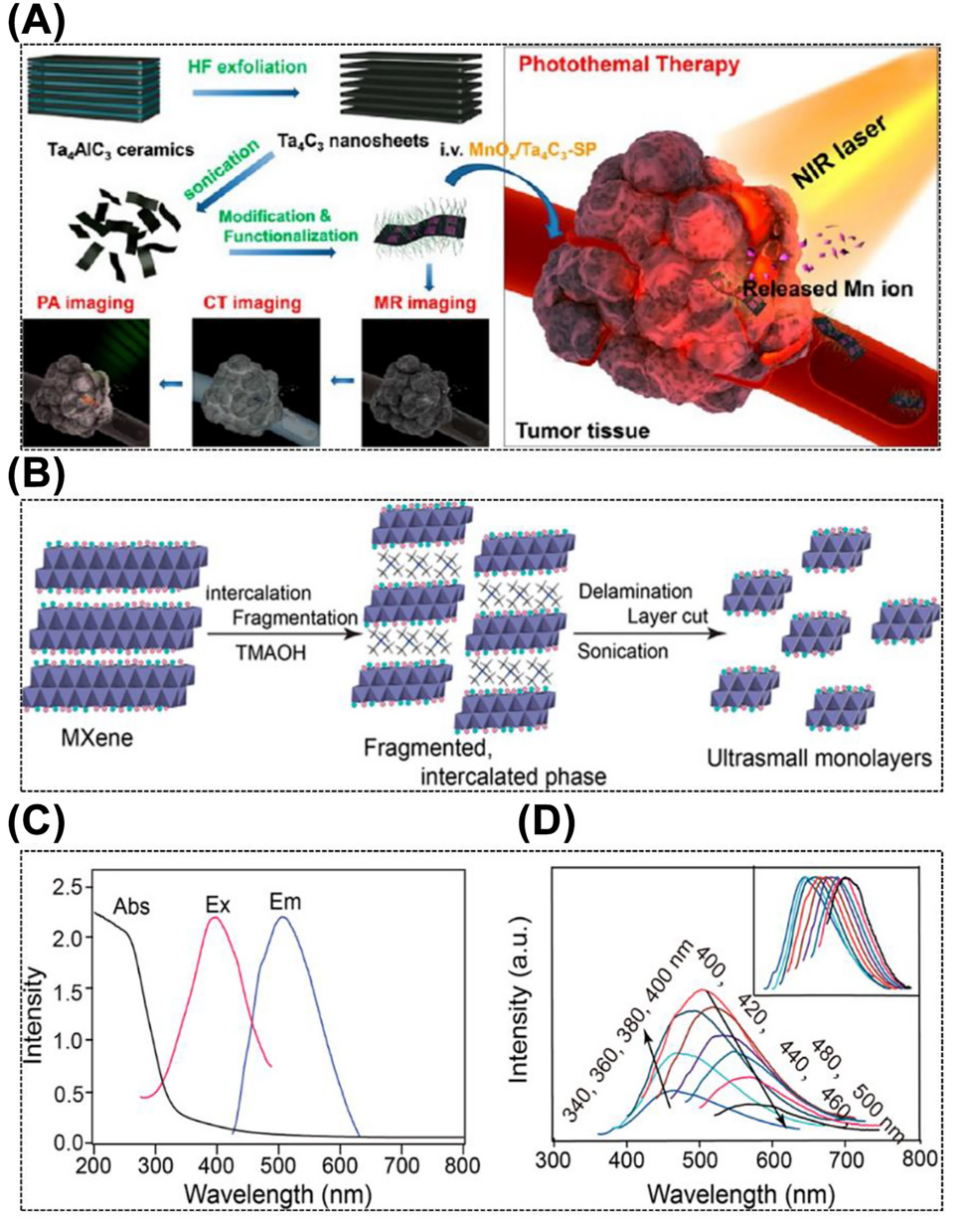
Photonic properties of MXene. (A) Schematic diagram of fabrication and surface modification process of MnOx/Ta_4_C_3_ and PTT guided by PA/CT/MR tri-modal imaging [24]. Copyright 2017, American Chemical Society. (B) Schematic diagram of the synthesis process for the preparation of ultrathin Ti_3_C_2_ flakes. (C) UV–vis absorption, emission, and excitation spectra of Ti_3_C_2_ sheets. (D) emission spectra at different excitation wavelengths from 340 to 500 nm. The inset in (D) shows the corresponding normalized emission spectra [28]. Copyright 2017, American Chemical Society.

The high potential of MXenes for photoacoustic (PA) imaging and photothermal therapy (PTT) at the first or second biological window is implied by their significant absorption properties throughout a broad spectral range from UV-visible to NIR (up to 1350 nm) [37]. The photothermal conversion efficiency of MXenes could reach roughly 50% under certain circumstances [37]. MXenes with highly metallic conductivities possess a unique advantage of serving as a substrate for surface-enhanced Raman spectroscopy, because of their intense localized surface plasmon resonance (LSPR) effect [24, 96] (Figure 3A). In addition, their hydrophilic and flexible surface is able to engage closely and steadily with the Raman Tags. Both of these factors contribute to the enhancement of the Raman scattering signal. Recently, the emission feature of luminescent MXene-based QDs has received significant interest [27]. Intriguingly, the luminescent MXene QDs were made by creating tiny dot-phase MXenes. With the aid of size-effect-induced quantum confinement and defect-induced luminescence, MXene QDs can produce specific emissions when excited at a given wavelength (mainly the UV-blue excitations currently) [27, 28] (Figure 3B–D). Probably due to the optical selection regarding various surface defects or sizes of the sampled ensemble, the synthesized Ti_3_C_2_ MXene QDs exhibit quantum dot excitation-dependent emission behavior comparable to that of graphene or carbon quantum dots. The fluorescent peaks increased from 460 to 580 nm following the shifting of excitation wavelength from 340 to 500 nm. To enable MXenes for widespread biomedical applications, modulating MXenes’ optical properties, including optical absorption, emission, and scattering, requires the creation of compounds with different compositions, structures, and surface terminations. However, there is still a lack of knowledge on the luminescence efficiency, emission color, and mechanism of MXenes.

### 2.5 Biosafety

The biodegradability, toxicity, and biocompatibility of 2D materials are essential to accelerate the process of clinical transformation. A series of paradigms confirmed that MXenes and their associated nanocomposites have high biocompatibility in biomedical applications. The activity of normal cells (MRC-5, MCF-10A, and HaCaT cell lines) was not significantly impacted by the presence of MXenes (Ti_2_N, Ti_2_C, and Ti_3_C_2_), and more than 70% of the cells survived even at MXene concentrations up to 500 μg/mL [97–99]. In contrast, the cytotoxic effect on tumor cells (A549, MCF-7, and A375 cells) was more than that on normal cells (MRC-5, MCF-10A, and HaCaT cells) [100]. In addition, Ti_2_AlN, Ti_3_AlC_2_, Ti_3_SiC_2_, and Mg/PLGA/Ti_3_C_2_ not only had no toxic effects on pre-osteogenic cells (CRL-2593 and MC3T3-E1 cells) in culture but also enhanced cell proliferation, indicating that MXenes possess a strong cellular affinity.

Several types of 2D MXenes (Ti_3_C_2_, Mo_2_C, and Nb_2_C) have been demonstrated to be biodegradable [92]. For instance, our team simulated the degradation properties of Nb_2_C-PVP nanosheets by utilizing human myeloperoxidase (hMPO), which produce reactive radical intermediates and hypochlorous acid to degrade carbon-based nanomaterials [37]. After adding hydrogen peroxide and hMPO, the suspension of Nb_2_C nanosheets became translucent and even almost disappeared after 24 h, suggesting that Nb_2_C MXene naturally has transcendental hMPO-responsive biodegradability. Moreover, blood biochemical parameters, hematological indexes and histopathological examinations showed that Nb_2_C-PVP nanosheets did not cause any apparent infection, inflammation, and hepatic and renal toxicities in mice within 28 days. The high biocompatibility of MXenes *in vitro* and *in vivo* with no harmful biodegradation in the safe dose range is the basis for the development of biological applications. The key challenge for continued biomedical research of MXenes will be to produce optimal performance while keeping safe levels.

## 3 Photonic biomedical applications of 2D MXenes

The distinctive photonic features of MXenes imply their high potential for use in numerous biological applications. Up to now, 2D MXenes and their composites have been used in the treatment of many diseases such as cancer, inflammation, brain diseases, kidney damage, viral infections, and cardiovascular diseases [92]. In this section, we will focus on the photonic biomedical applications of MXenes, such as antibacteria, bioimaging, therapies, and theranostics.

### 3.1 Photonic antibacterial activity

**Figure 4:**
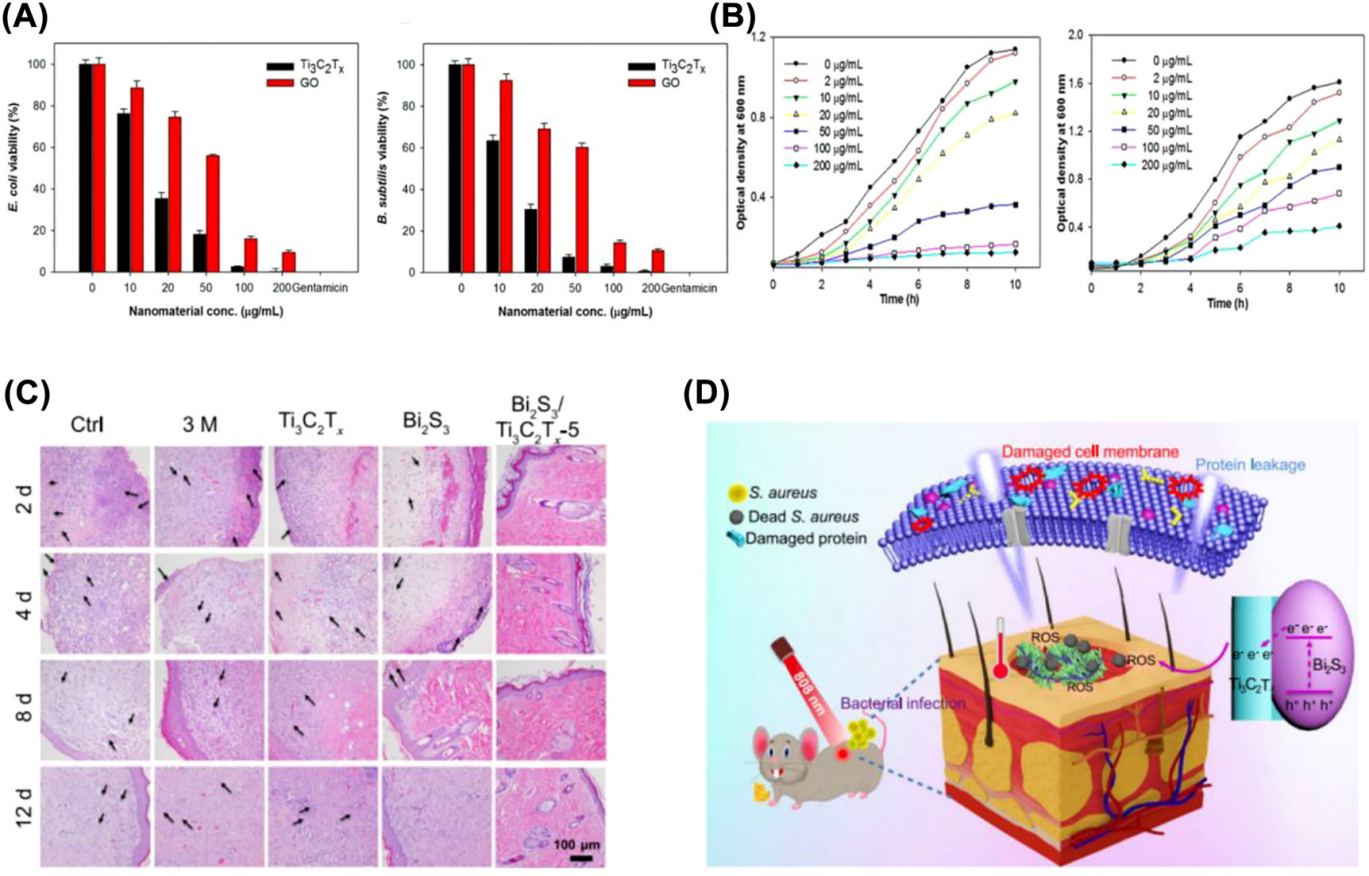
Antibacterial properties of MXene. Determination of cell viability of (A) *E. coli* and (B) *B. subtilis* treated with Ti_3_C_2_T_*x*_ and GO in aqueous suspension. (B) OD regeneration curves of (A) *E. coli* and (B) *B. subtilis* in LB broth after treatment with various concentrations of Ti_3_C_2_T_*x*_ [18]. Copyright 2016, American Chemical Society. (C) HE staining of wounds. (D) Schematic diagram of Bi_2_S_3_/Ti_3_C_2_T_*x*_ Schottky action and antibacterial mechanism of Bi_2_S_3_/Ti_3_C_2_T_*x*_ irradiated at 808 nm [19]. Copyright 2021, Springer Nature.

MXenes with ultrathin layered morphology, amazing physicochemical properties, remarkable photothermal properties, and excellent biocompatibility exhibit intriguing photonic antibacterial effects. The mechanism of action can be summarized in the following four aspects: (1) monolayer MXene can adsorb microorganisms and lead to bacterial membrane disruption due to the exposure of cells to its acute edges; (2) hydrogen bonds between the oxygen-containing groups of MXene nanosheets and cell membrane lipopolysaccharide chains prevent bacterial growth by inhibiting nutrient uptake; (3) the negative charge on the surface of MXene nanosheets and their high hydrophilicity can enhance the contact between bacteria and the membrane surface, leading to inactivation of adherent bacteria; (4) MXene nanosheets can also react with some molecules in the cytoplasm and cell wall of microorganisms, resulting in the destruction of cell structure and subsequent cell death [61]. For instance, similar to semiconductors that produce negative electrons and positive holes, Ti_3_C_2_T_*x*_ MXene can use reactive metal-F pairs to transfer reactive electrons to the surrounding cell membrane under excitation by light. As a result, cells were killed in a manner akin to type I photodynamic treatment (PDT), thereby inhibiting the growth of Gram-negative *Escherichia coli* (*E. coli*) and Gram-positive *Bacillus subtilis* (*B. subtilis*) [18]. Additionally, it has been discovered that Ti_3_C_2_T_*x*_ MXene nanosheets show a significant concentration-dependent bactericidal ability and outperform GO in terms of antibacterial activity (Figure 4A and B). Subsequently, in 2017, Rasool et al. fabricated micrometer-thick Ti_3_C_2_T_*x*_ MXene nanosheets coated with polyvinylidene fluoride (PVDF) support, demonstrating their extraordinary antibacterial properties under the synergistic effect of Ti_3_C_2_T_*x*_ MXene nanosheets and anatase TiO_2_ nanocrystals formed on sharp edges [101]. According to these findings, Pandey et al. further synthesized Ag NPs-modified Ti_3_C_2_T_*x*_ MXene nanosheets to act as an ultrafast water purification membrane owing to their excellent anti-biofouling property [21]. In addition, Lv et al. assembled Ag NPs on PEG-modified Ti_3_C_2_T_*x*_ MXene to improve the stability and sterilization of the material system in an aqueous solution [20]. The antibacterial test results showed that as-synthesized MXene-based hybrid antibacterial system (M-HAS) has remarkable antibacterial activity against *E. coli* and *Staphylococcus aureus* at 660 nm laser.

More recently, in consideration of the increasing antibiotic resistance, Li et al. prepared an eco-friendly interfacial Schottky junction of Bi_2_S_3_/Ti_3_C_2_T_*x*_ caused by the contact potential difference between Ti_3_C_2_T_*x*_ and Bi_2_S_3_ (Figure 4D) [19]. The photocatalytic activity of Bi_2_S_3_/Ti_3_C_2_T_*x*_ significantly enhanced the production of reactive oxygen species (ROS) under near-infrared radiation at 808 nm Bi_2_S_3_/Ti_3_C_2_T_*x*_ nanocomposites are able to kill 99.86% of *S. aureus* and 99.92% of *E. coli* under NIR irradiation within 10 min, as demonstrated by live/dead fluorescence staining, SEM, and spread plate. Hematoxylin and eosin (H&E) staining also demonstrated that the Bi_2_S_3_/Ti_3_C_2_T_*x*_ Schottkyte nodules can effectively promote wound healing (Figure 4C). Likewise, Ti_3_C_2_T_*x*_–Au was created by Yu et al. to combat *E. coli* and *B. subtilis* [102]. *E. coli* only had a 0.75% survival rate under 808 nm laser irradiation, while *B. subtilis* completely perished. Furthermore, using a Nb_2_C MXene titanium plate as the foundation, Yang et al. created a therapeutic implant with multimodal anti-infection properties [22]. This new type of MXene is able to destroy biofilms by directly killing bacteria by down-regulating bacterial energy metabolism pathways, inhibiting biofilm formation, and augmenting as-formed biofilm detachment. This clinical implant enhanced bacterial sensitization with the aid of photothermal transduction, enabling a decrease in the temperature required for bacterial elimination as well as a reduction in probable healthy tissue injury. Aside from killing bacteria *in vivo*, the medical implant based on Nb_2_C MXene can also mitigate the pro-inflammatory response by removing excess ROS from the infectious microenvironment, thereby promoting tissue remodeling and angiogenesis. In addition, V_2_C MXene was created by Zada et al. with a favorable structure, significant near-infrared (NIR) absorption, and exceptional photothermal conversion competency, making it a prominent bactericidal photothermal agent [23]. The antibacterial efficiency evaluation shows that the V_2_C MXene has a substantially higher ability than previous report for Ti_3_C_2_, Ta_4_C_3_ and Nb_2_C MXenes to kill Gram-negative *E. coli* and Gram-positive *S. aureus* under NIR laser irradiation for 5 min.

MXenes with varied physicochemical features exhibit a range of antibacterial capabilities as a result of their chemical behavior, which is primarily influenced by the types of transition metals and surface-terminating functional groups. For example, stoichiometry can influence the antibacterial effect of MXenes, as observed by Jastrzebska et al. [103]. They found that Ti_3_C_2_T_*x*_ MXene can inhibit bacterial growth, which is consistent with previous studies. Ti_2_CT_*x*_ MXene, however, has a limited ability to prevent bacterial growth. Based on the X-ray photoelectron spectroscopy (XPS) results, it appeared that the surface chemistry of Ti_2_CT_*x*_ and Ti_3_C_2_T_*x*_ MXene was similar, therefore, the authors proposed that the antimicrobial activity of MXenes with particular stoichiometries is attributed to structural differences at the atomic scale. Especially, Shamsabadi et al. confirmed that the antibacterial activity of MXene nanosheets was determined by size and exposure time *via* quantifying bacterial species using fluorescence imaging, flow cytometry, and complementing techniques [104]. The smaller nanosheets showed satisfactory antibacterial effects against both Gram-positive and Gram-negative bacteria. As a result, precise control of ultrathin MXene nanosheets is crucial to maximizing their potential for use in antimicrobial applications.

### 3.2 Photonic bioimaging

Aside from antibacterial property, MXenes are highly sought after in bioimaging due to their distinctive photonic characteristics including powerful X-ray attenuation, prominent photothermal conversion capability, and remarkable fluorescence quenching. Up to now, MXenes have received substantial research in a variety of domains, including photoacoustics, fluorescence, computed tomography, magnetic resonance, thermal infrared imaging [100], etc. In this section, we primarily concentrate on the application of MXenes in the area of photonic bioimaging, such as PA imaging, fluorescent imaging, and multiple photonic imaging.

#### 3.2.1 PA imaging

PA imaging, as one of the emerging imaging techniques, is a specific biomedical imaging modality that has been used in disease monitoring, drug delivery, and surgical guidance [105]. As a rapidly developing technology with high contrast and large penetration depth, it combines the outstanding contrast of optical biomedical imaging with the extremely deep tissue penetration capability of ultrasound imaging, overcoming the shortcomings of conventional optical imaging to produce label- and speckle-free images, especially in deep biological tissues [106]. PA imaging contrast agents (CAs) with outstanding photothermal conversion are indispensable for obtaining significant PA signals, therefore, further studies of CAs are needed to improve the resolution and sensitivity of this technique. In general, MXenes nanosheets exhibit a semi-metallic-like energy band structure that readily induces the LSPR effect, enabling them to exhibit excellent absorption properties and high conversion efficiency under broad spectra light irradiation [35]. Consequently, MXenes including Ti_3_C_2_ and Ta_4_C_3_ could serve as effective CAs for PA imaging [24, 25]. Especially, deep-tissue PA imaging is particularly feasible with MXenes because of their wide and dense NIR absorption spectra.

**Figure 5:**
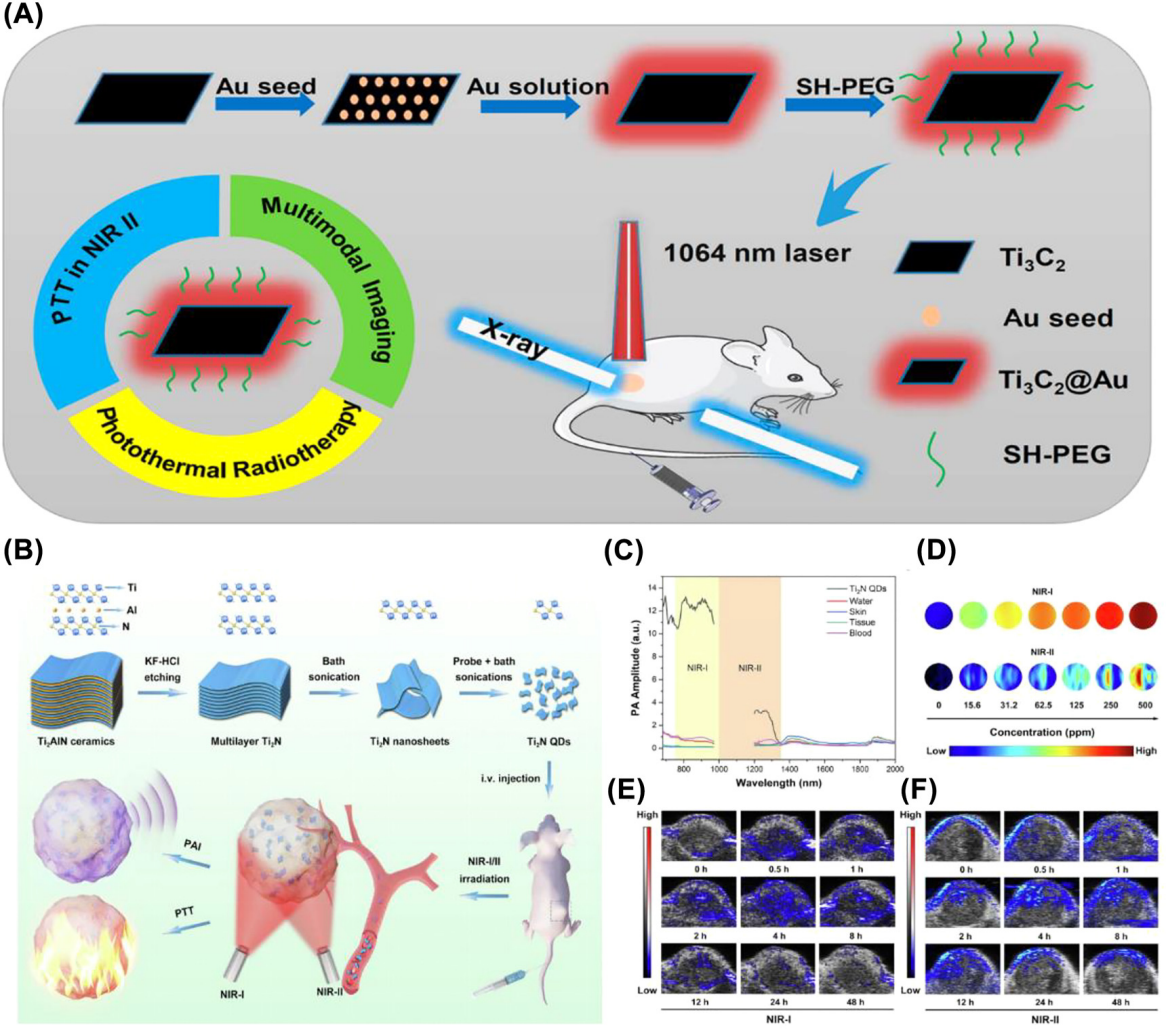
PA imaging of MXene. (A) Schematic diagram of Ti_3_C_2_@Au synthesis and PA/CT dual-modality imaging-guided treatment [47]. Copyright 2019, American Chemical Society. (B) Schematic diagram of Ti_2_N QDs synthesis and the role of PTT guided by PA imaging in NIR-I/II biological windows. (C) PA signals of Ti_2_N QDs in NIR-I/II regions by taking different samples (water, skin, tissue, and blood of mice) as the control group. (D) *In vitro* PA images of Ti_2_N QDs at various concentrations (from 0 to 500 ppm) irradiated by 808 and 1280 nm laser. (E–F) *In vitro* PA images in tumor tissue after intravenous injection [26]. Copyright 2020, Elsevier.

So far, several kinds of MXenes including Ti_3_C_2_, Ta_4_C_3_, Ti_3_N, V_2_C, and Nb_2_C have been applied for *in vitro* and *in vivo* PA imaging under the NIR laser irradiation [100]. On top of that, we have fabricated the MnO_*x*_/Ta_4_C_3_ composite nanosheets, which can achieve multiple imaging-guided photothermal tumor ablation and tumor growth inhibition [24]. Furthermore, Tang et al. constructed Ti_3_C_2_@Au nanocomposites with remarkable photothermal conversion, as shown in Figure 5A, which showed stronger photoacoustic signals than the same concentration of Ti_3_C_2_ alone for imaging and quantitative measurements from 680 to 970 nm [47]. More recently, Shao et al. have prepared Ti_2_N QDs with a diameter of about 5 nm that exhibit high photothermal conversion efficiency in both NIR-I and NIR-II laser excitation (Figure 5B–D) [26]. As a result of the characteristic enhanced permeability and retention (EPR) effect, Ti_2_N QDs quickly accumulated, which quickly increased the PA signal in the tumor tissue following the initial phase of circulatory delivery (Figure 5E and F).

It is noted that the development of MXenes for PA imaging is encouraged by their superior optical qualities, distinctive biological characteristics, and top performance even if the use of MXenes in PA imaging is still in the research stage.

#### 3.2.2 Fluorescent imaging

**Figure 6:**
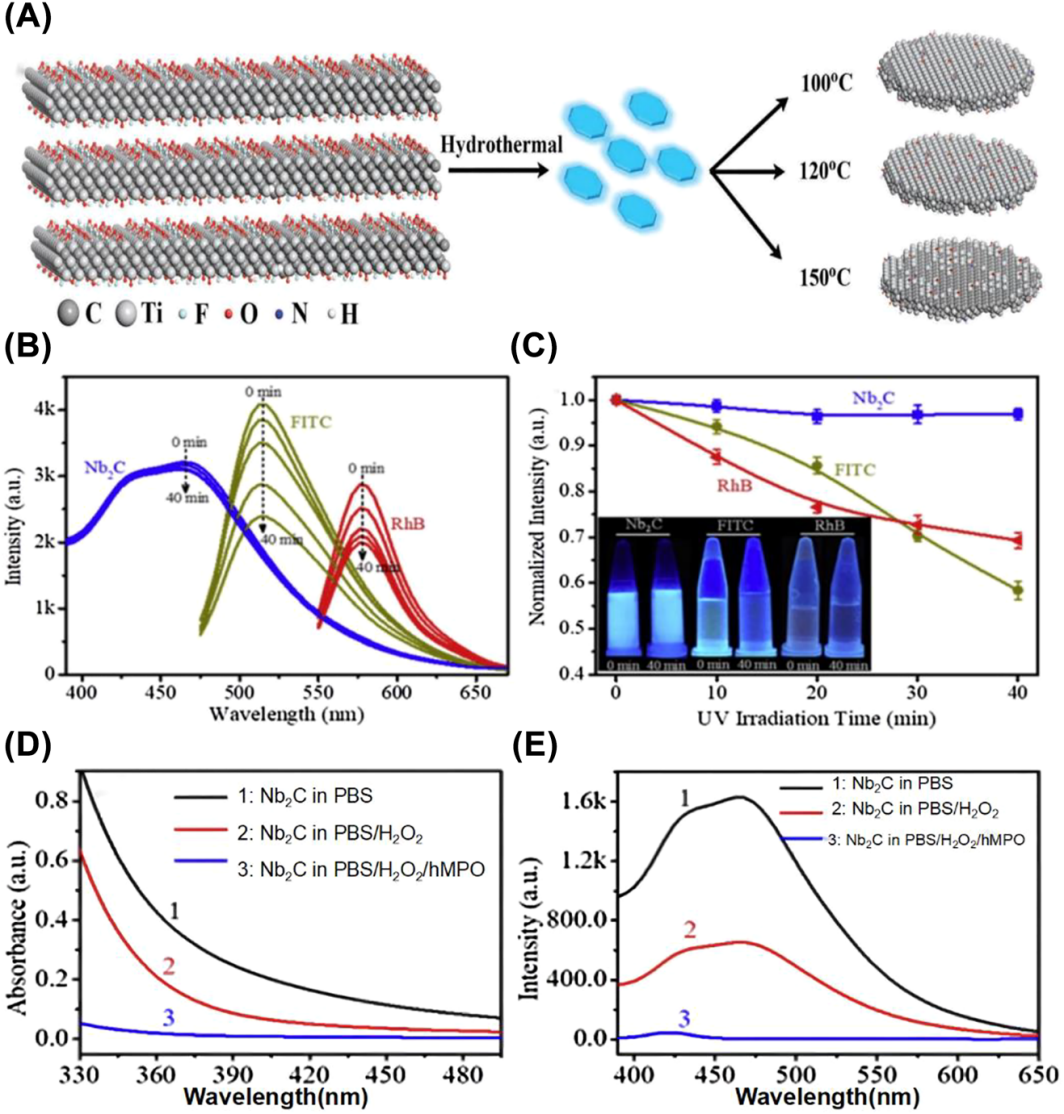
Fluorescence imaging of MXene. (A) Diagram of the preparation of Ti_3_C_2_ QDs [27]. Copyright 2017, Wiley-VCH. (B–C) PL emission spectra (B) of Nb_2_C QDs and normalized PL intensity (C) corresponding to three aqueous solutions (Nb_2_C QDs, FITC, and RhB) at different durations of irradiation with a 350 W xenon lamp. (D–E) UV spectra (D) and PL emission spectra (E) of Nb_2_C QDs after different degradation treatments [30]. Copyright 2018, Elsevier.

Fluorescence imaging is frequently employed in biomedical research due to its excellent imaging sensitivity and straightforward operation. In the past few years, 2D biomaterials and their corresponding quantum dots (QDs) possessing tunable wavelength, high photostability, and desirable quantum yields have attracted considerable attention in fluorescence imaging [52]. As demonstrated by Xue et al., Ti_3_C_2_ QDs with defect-induced luminescence and size-effect induction formed by hydrothermal fracture can be effectively used for cellular fluorescence imaging [27] (Figure 6A). Similarly, other MXene QDs including Nb_2_C [30, 31] and V_2_C [107] can also serve as imaging contrast agents for bioimaging.

As previously mentioned, Xue et al. fabricated Ti_3_C_2_ MXene QDs by a simple hydrothermal method and successfully tailored their size by adjusting the reaction temperature [29]. They found that the MXenes are capable of readily entering cells for *in vitro* bioimaging by endocytosis without causing genetic damage. Wang et al. created the Ti_3_C_2_ QDs by simultaneously stacking cleavage and layer cutting in tetramethylammonium hydroxide [28]. The resulting monolayer Ti_3_C_2_ QDs exhibit distinct and tunable fluorescence with powerful photoluminescence and excitation-dependent behavior. Importantly, the authors suggested that additional ultra-small MXene nanosheets, such Nb_2_C or Ti_2_C, can also be obtained using this all-encompassing and gentle method, which broadens the use of MXene materials in optical-related domains. Except for Ti_3_C_2_ QDs, Yang et al. have effectively employed Nb_2_C QDs for fluorescence sensing and fluorescent imaging of heavy metal ions. They successfully produced Nb_2_C QDs with high photostability, biodegradability, and strong resistance to photobleaching (Figure 6B and C) [30]. Nb_2_C QDs can be degraded by human myeloperoxidase (hMPO) (Figure 6D and E). As another example, Yan et al. have prepared nitrogen and sulfur co-doped Nb_2_C MXene QDs with significant green fluorescence through the hydrothermal method [31]. The obtained Nb_2_C MXene QDs possessed the characteristics of anti-photobleaching, high dispersion stability, and photostability, which led to their successful application in copper ion detection with a detection limit of 2 μmol/L and Caco-2 cell imaging.

#### 3.2.3 Multiple bio-imaging

**Figure 7:**
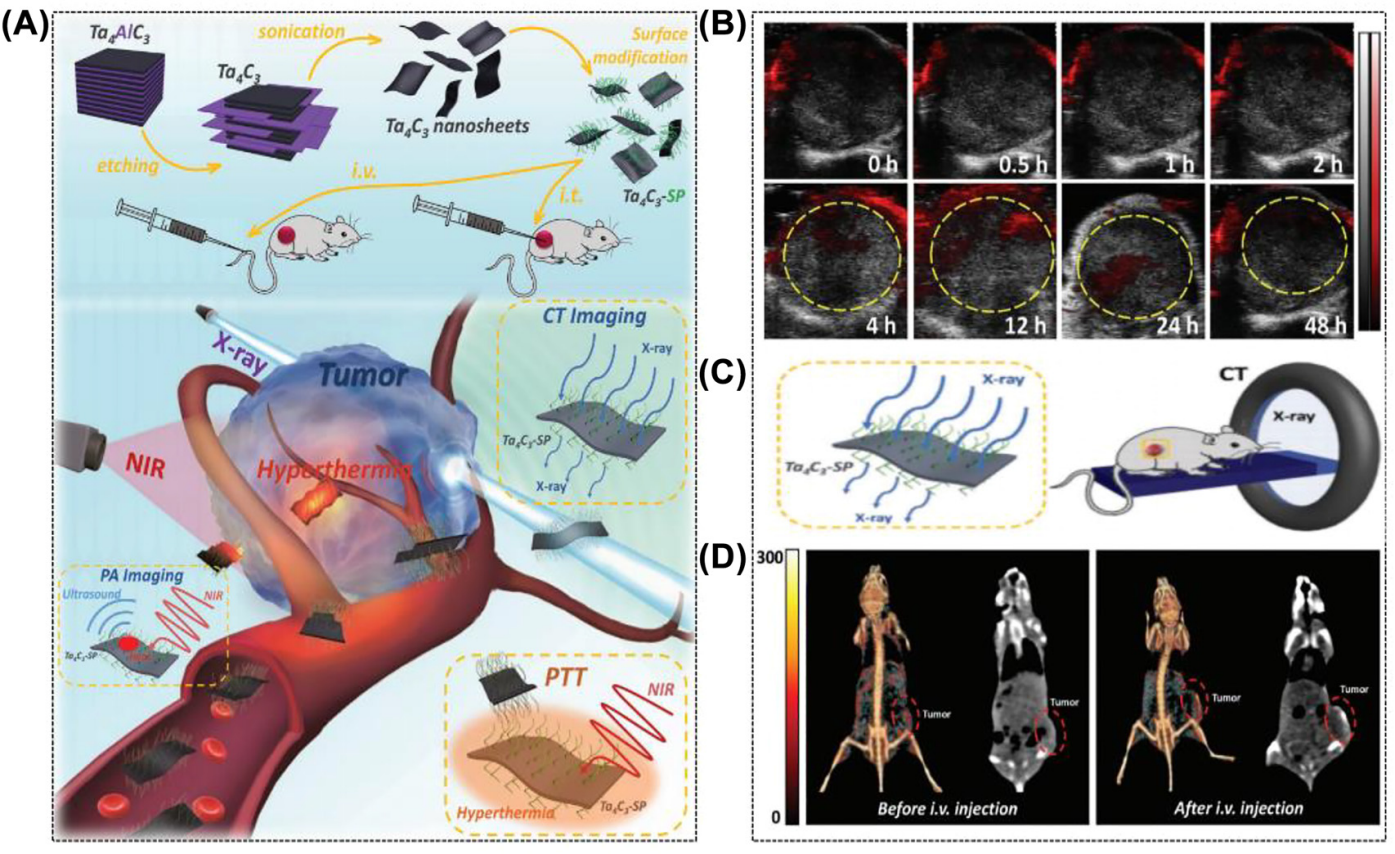
Multiple imaging capabilities of MXene. (A) Schematic representation of Ta_4_C_3_ nanosheet synthesis and *in vivo* PA/CT dual-mode imaging-guided photothermal tumor ablation. (B) PA images of tumor tissues after 0, 0.5, 1, 2, 4, 12, 24 and 48 h injections. (C) Schematic illustration of Ta_4_C_3_-SP enabling *in vivo* CT imaging. (D) *In vivo* 3D reconstructed CT (left) and CT comparison (right) images of mice before and after 24 h intravenous injection [33]. Copyright 2018, Wiley-VCH.

Generally speaking, the MXene family with high atomic number metallic elements, such as tantalum (Ta) (*Z* = 73) and tungsten (W) (*Z* = 74), is a promising candidate contrast agent for CT imaging with exceptional X-ray attenuation. For instance, our group has successfully synthesized 2D ultrathin Ta_4_C_3_ MXene nanosheets with SP modification through HF etching and probe sonication, and they are successfully applied in dual-mode PA/CT imaging for the first time by taking advantage of their strong X-ray attenuation ability and high NIR optical absorbance (Figure 7) [32]. More importantly, greater Ta concentrations resulted in a higher Hounsfield unit (HU) value for Ta_4_C_3_ MXene nanosheets than the clinically utilized iopromide. As another paradigm, our group reported W_1.33_C-based i-MXene nanosheets with ordered exfoliation *via* selective etching of Al and yttrium (Y) elements [33]. Thanks to the strong X-ray attenuation capability of W, the outstanding absorbance of the NIR region, and the excellent photothermal conversion capability, the CT/PA/photothermal tri-mode imaging is successfully achieved. Similarly, experimental results showed that both PA image signals and the HU values of CT images increased linearly with increasing W_1.33_C MXene concentration. The photoacoustic signal intensity was 10 times greater after the medicine was injected intravenously for 4 h, and the CT signal intensity of the tumor tissue was dramatically improved from 37.5 to 98.2 HU.

**Figure 8:**
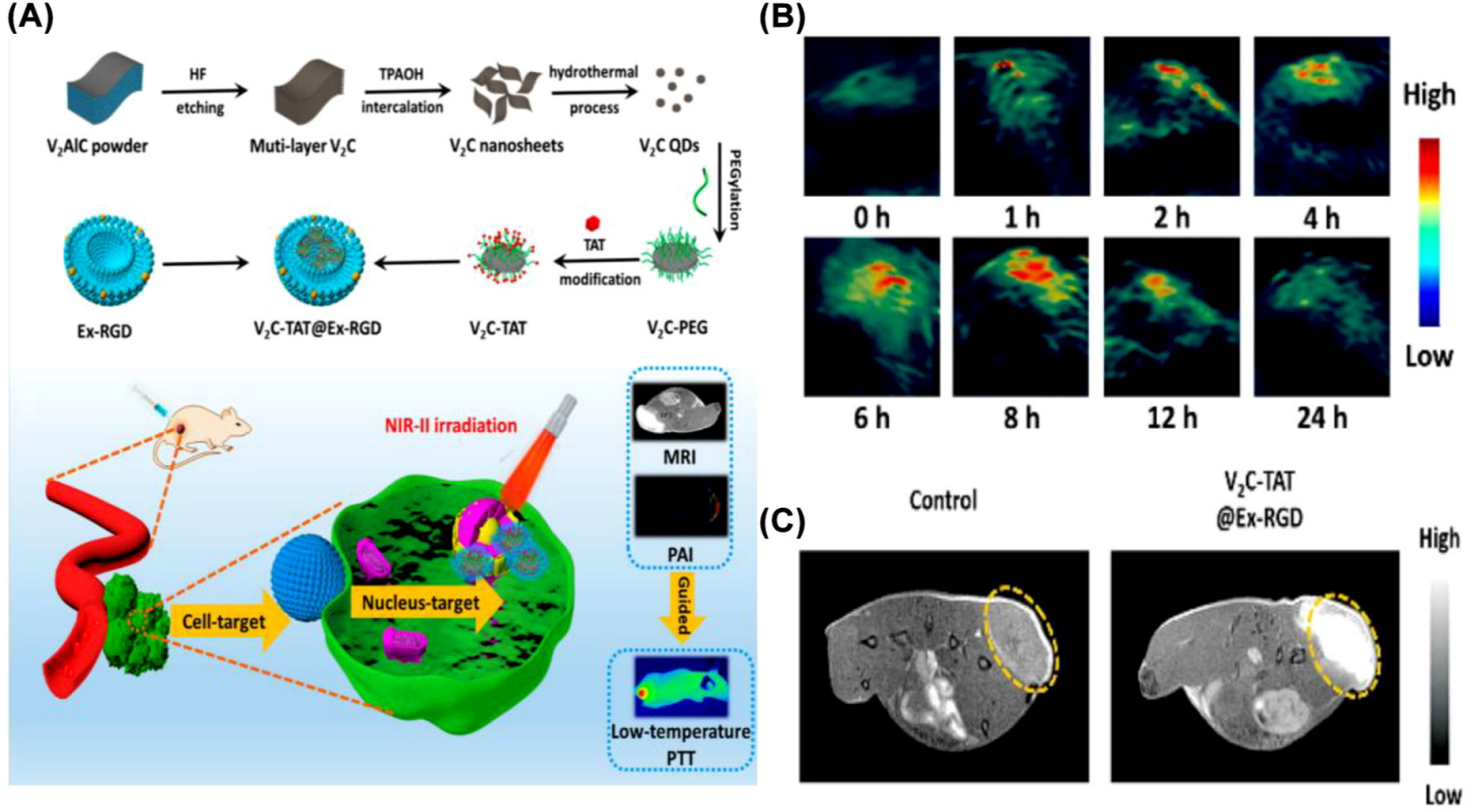
Multiple imaging capabilities of MXene. (A) Scheme of the synthesis and surface modification of V_2_C-TAT@Ex-RGD and multimodal imaging in NIR-II biological window combined with low-temperature PTT. (B) *In vivo* PA images of mice at different times after *i. v.* injection of V_2_C-TAT@Ex-RGD (V_2_C-TAT, 10 mg/kg). (C) MR images of mice after intravenous injection of PBS (left) and V_2_C-TAT@Ex-RGD (right) [34]. Copyright 2019, American Chemical Society.

Except for CT-related photonic imaging, Cao and co-workers fabricated V_2_C QDs with significant MR imaging capability packed with RGD modification [34]. The as-prepared nanocomplexes had multimodal imaging capabilities including PAI, fluorescence imaging, and MR imaging (Figure 8). In addition, the V_2_C-TAT@Ex-RGD also exhibited good biocompatibility, long cycle time, and endosome escape capability. Furthermore, MnO_*x*_/Ta_4_C_3_ and MnO_*x*_/Ti_3_C_2_ nanocomposites with multimodal imaging properties have been constructed by inducing redox reactions between oxidized MnO_*x*_ and the surface groups of MXenes [24, 25]. MnO_*x*_/Ti_3_C_2_ composite MXenes have a strong photothermal conversion, which makes them desirable for PA imaging. Mn, a regularly utilized paramagnetic agent, is also present in these materials, which allows them to accomplish T_1_-weighted MR imaging. With regard to MnO_*x*_/Ta_4_C_3_ composite nanosheets, PA, CT, and MR tri-mode imaging are made possible by their excellent photothermal conversion performance, potent X-ray attenuation ability of Ta, and T_1_-weighted MRI capability of integrated Mn.

### 3.3 Photonic therapeutic applications

2D multifunctional MXene and its composites have found extensive use in biomedicine thanks to their varied chemical structures and distinctive photonic features. In this section, photonic therapeutic applications of MXenes, including PDT, PTT, photonic synergistic therapy, and theranostics, are discussed in detail with the selected paradigms.

#### 3.3.1 Photodynamic therapy (PDT)

PDT is a technique in which localized tumor tissue is exposed to particular light wavelengths to activate multifunctional photosensitizers that release ROS to kill cancer cells [108]. PDT provides some noteworthy advantages over traditional tumor therapy methods, such as weak aggressiveness, non-toxic reproducibility, low side effects, remarkable treatment effects, and low long-term morbidity. Basically, PDT uses light with specific wavelengths to activate multifunctional photosensitizers that aggregate at the tumor site, and the activated photosensitizers transfers energy to surrounding oxygen molecules, leading to the production of ROS and oxygen reduction, thereby oxidizing the biomolecules that cause cancer cell death [108, 109]. Owing to the distinctive optoelectronic properties and electronic structure of MXene, it is suggested by theoretical studies that MXene itself can be employed as a photosensitizer for PDT [39].

In 2017, the Ti_3_C_2_ MXene was firstly demonstrated to produce ROS upon irradiation with specific light wavelengths, implying its great possibility as a photosensitizer for PDT [44]. Experiments showed that this multifunctional Ti_3_C_2_ MXene nanoplatform modified by doxorubicin (DOX) and hyaluronic acid (HA) exhibits excellent photothermal conversion and sufficient ^1^O_2_ generation under 808 nm laser irradiation. It has been found that using 1,3-diphenylisobenzofuran (DBPF) as an oxygen detector, the absorption peak of DPBF at 420 nm decreased significantly after 10 min of laser irradiation, indicating a significant amount of ROS production. This might be a result of the LSPR effect brought on by its large specific surface area and the photoexcited electron transfer effect. Subsequently, Zhang et al. constructed Mo_2_C nanospheres with negligible blood and tissue toxicity and excellent biocompatibility for cancer therapy [43]. Similarly, the absorption peak of DPBF in aqueous solution decreased after laser irradiation, confirming the generation of ROS. Due to the disappointing results of single-modality therapy, PDT is less frequently used alone to treat tumors; instead, it is frequently used in conjunction with chemotherapy and PTT for photonic theranostics.

#### 3.3.2 Photothermal therapy (PTT)

PTT is a strategy of converting photonic energy into thermal energy enabled by the photothermal transduction agents at the tumor site, thus producing heat to cause cell death. Since the original discovery of PTT, it has received significant interest because of its ease of surgery, short treatment time, low invasiveness, few side effects, and low recurrence rate [92]. Therefore, PTT can be a distinct alternative or complementary approach to conventional cancer treatments.

Generally, the photothermal agent and external light are the two key contributors to the PTT effect. For photothermal agents, the light absorption capacity, revealed by the extinction coefficient (*ε*), and the photothermal conversion efficiency (*η*), which measures their capability to convert light into heat, are two crucial characteristics that mainly affect their PTT results [35]. Typical 2D MXenes, including Ti_3_C_2_ [35], Ta_4_C_3_ [32], Nb_2_C [37], Mo_2_C [40], V_2_C [49], and Ti_2_C [42], have been shown to feature remarkable photothermal properties and are frequently utilized in PTT *in vivo* and *in vitro*.

**Figure 9:**
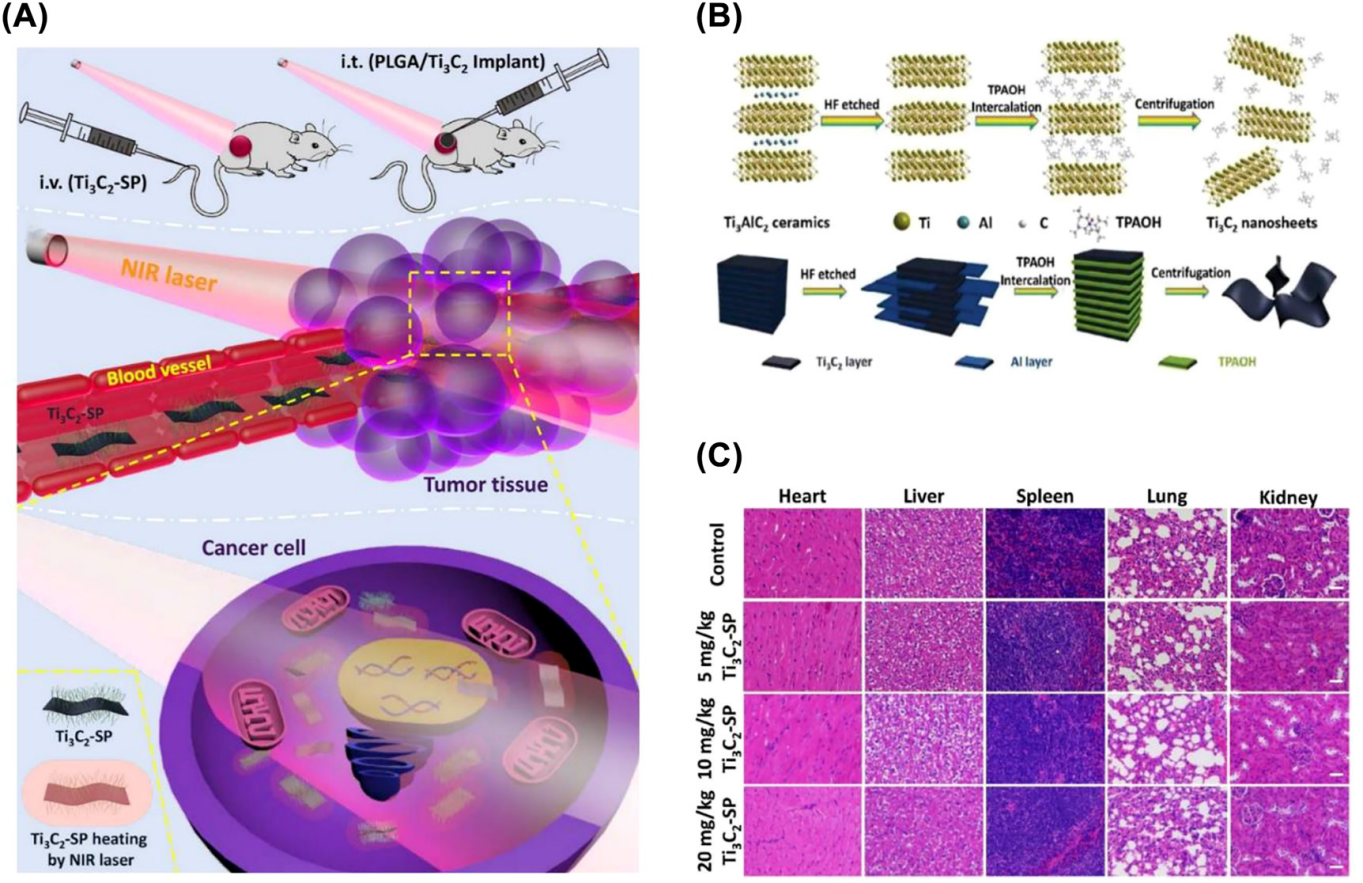
MXene for PTT. (A) Schematic representation of treatment with intravenous injection of Ti_3_C_2_-SP and intratumoral injection of PLGA/Ti_3_C_2_ implants under NIR laser irradiation. (B) Schematic illustration of the synthesis process of Ti_3_C_2_ nanosheet. (C) H&E staining of major organs (heart, liver, spleen, lung and kidney) in the control group and three treatment groups with different Ti_3_C_2_-SP doses (5, 10, and 20 mg/kg, respectively) [35]. Copyright 2017, American Chemical Society.

**Figure 10:**
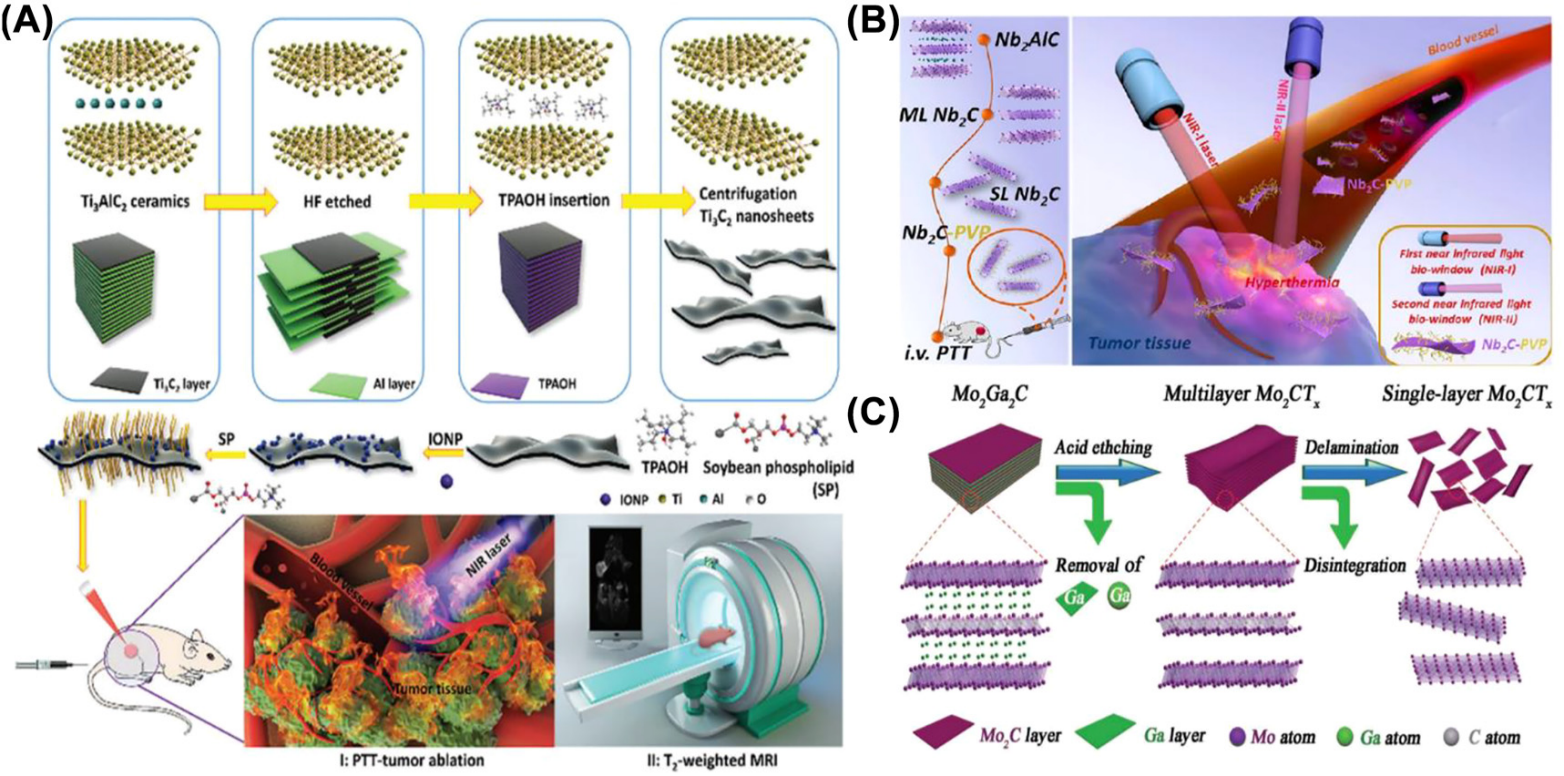
MXene for PTT. (A) Schematic representation of the exfoliation and surface modification process of magnetic 2D Ti_3_C_2_-IONPs-SPs nanosheet and its PTT under T_2_-weighted MRI guidance [113]. Copyright 2019 The Royal Society of chemistry. (B) Schematic representation of the synthesis process of Nb_2_C and *in vivo* PTT in NIR-I and NIR-II biological windows [37]. Copyright 2017 American Chemical Society. (C) Schematic representation of the preparation of Mo_2_C MXenes [40]. Copyright 2018 John Wiley and Sons.

In 2016, our team synthesized the SP-modified Ti_3_C_2_ MXene nanosheets by a classical two-step exfoliation strategy that involved HF etching and TPAOH intercalation for the first time (Figure 9) [35]. The biosafe Ti_3_C_2_ exhibited photothermal conversion of up to 30.6% when exposed to a NIR laser irradiation (808 nm), which was significantly better than the majority of currently available photothermal materials like gold nanorods (21%) [110], FeS nanosheets (15.5%) [111] and Bi_2_S_3_ nanosheets (20.5%) [112]. Subsequently, we synthesized SP-modified MnO_*x*_/Ti_3_C_2_ nanocomposites through *in situ* growth for PTT against cancer [25]. The results showed that the tumor temperature in the MnO_*x*_/Ti_3_C_2_-SP NIR group increased from about 25 °C to 60 °C at 10 min of laser irradiation, which could entirely lead to the destruction of cancer cells. In addition, our team constructed superparamagnetic 2D Ti_3_C_2_ MXenes with high photothermal conversion efficiency (48.6%), which ensured effective photothermal killing of cancer cells and ablation of tumor tissue as demonstrated by *in vitro* and *in vivo* experiments (Figure 10A) [113]. The experimental results demonstrated the high biocompatibility of the magnetic Ti_3_C_2_-IONPs MXene composites as manufactured indicating their potential for further clinical applications.

As a PTT agent with high photothermal conversion efficiency, Ta_4_C_3_ has made fast progress in tumor therapy. The 2D SP-modified Ta_4_C_3_ nanosheets with 44.7% photothermal conversion efficiency exhibited high *in vivo* photothermal ablation of tumor xenografts [32], which were synthesized by two-step liquid phase stripping. More importantly, both *in vitro* cellular and *in vivo* mouse experiments demonstrated that the SP-modified Ta_4_C_3_ nanosheets have no discernible toxicity. On this basis, we applied MnO_*x*_/Ta_4_C_3_ composite nanosheets (*η* = 34.9%) fabricated by activating redox reactions on the surface of Ta_4_C_3_ for multi-target photothermal tumor ablation [24]. Under 808 nm laser irradiation, the MnO_*x*_/Ta_4_C_3_ exhibited concentration/laser power-dependent photothermal properties and significantly inhibited tumor growth. Additionally, our group reported the Ta_4_C_3_ nanosheets (*η* = 32.5%) modified with Fe_3_O_4_ nanoparticles (IONPs) and SP applied for multiple imaging-guided tumor therapy [36]. Experimental results showed that Ta_4_C_3_-IONP-SPs composite nanosheets completely eradicate tumors without recurrence, which further broadens the biomedical applications of MXene nanoplatform.

Since the original discovery of Nb_2_C, surface engineering has played an important factor in determining how it is used in the biological field. Nb_2_C nanosheets prepared by a two-part liquid exfoliation method showed high photothermal performance in the first and second biological windows with photothermal conversion efficiencies of 36.4 and 45.65%, respectively [37]. PVP-modified Nb_2_C was capable of effective *in vivo* photothermal ablation of tumor xenografts in the first and second biological windows, as shown by both *in vitro* cellular investigations and *in vivo* experiments. It also exhibited high biocompatibility and minimal toxicity (Figure 10B). Moreover, our team created a “therapeutic mesoporous” layer on the surface of 2D Nb_2_C MXenes using multifunctional sol–gel chemistry [38]. Nb_2_C MXenes exhibited a photothermal conversion capacity of 28.6% in the NIR-II biological window and performed strong inhibitory effects on both U87 brain cancer cells and subcutaneous tumors in nude mice.

More recently, we reported the construction of emerging PVA-modified 2D Mo_2_C MXene (Figure 10C) [40]. The Mo_2_C-PVA nanosheets had a wide absorption band of NIR in both I and II regions, as well as a desirable photothermal conversion efficiency (24.5% for NIR I and 43.3% for NIR-II), which could allow for effective photothermal tumor ablation. In addition, Zhang and his co-worker prepared 50 nm Mo_2_C nanospheres with excellent biocompatibility and minimal toxicity for both tumor treatment and imaging [43]. The photothermal conversion efficiency of 24.95% indicated the significant photothermal tumor ablation effect of Mo_2_C MXene.

**Figure 11:**
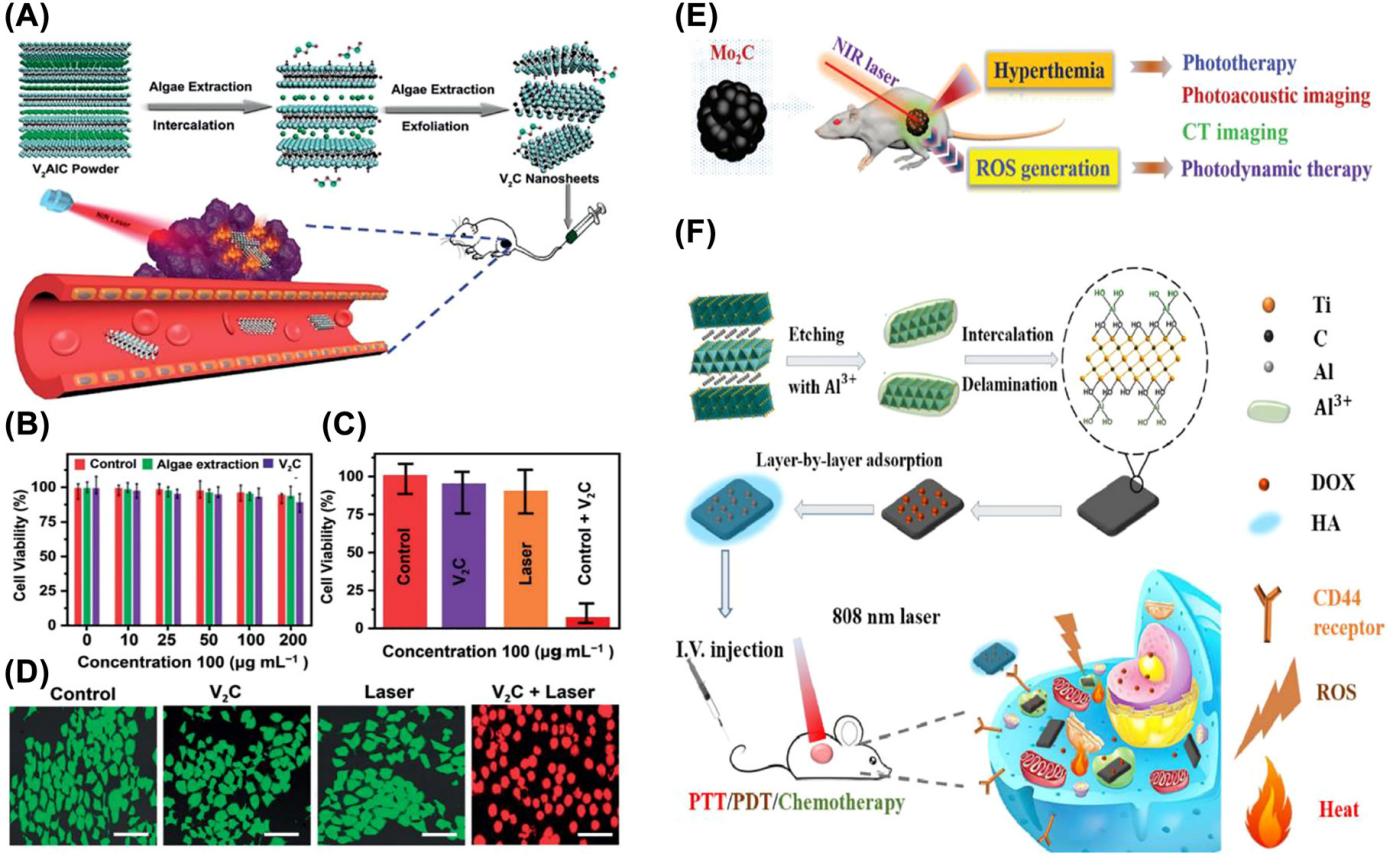
MXene for PTT. (A) Schematic diagram of the synthesis of V_2_C nanosheets by algae extraction and their application in tumor ablation. (B) Cytotoxicity analysis of MTT after control (PBS pH 7.4), algae extraction, and V_2_C treatment. MTT (C) and Calcein AM/PI double staining (D) cell viability analysis after different treatments (control, laser only, V_2_C only, and V_2_C + laser) [49]. Copyright 2020, Wiley-VCH. (E) Schematic diagram of the Mo_2_C nanosphere-mediated tumor treatment protocol [43]. Copyright 2019, The Royal Society of Chemistry. (F) Schematic illustration of the synthesis process of Ti_3_C_2_ nanosheet and multimodal tumor therapy [44]. Copyright 2017, American Chemical Society.

Two other MXene materials, V_2_C and Ti_2_C, are thought to be viable photothermal agents for PTT to treat cancer because of their high photothermal conversion efficiency. Zada et al. constructed V_2_C nanosheets using the algae extraction method for the insertion and stacking of the V_2_AlC MAX phase bulks (Figure 11A–D) [49]. The photothermal conversion efficiency of this material was 48%, which is on par with other top photothermal agents including carbon-based materials and gold-based nanostructures. This work not only demonstrated the strong tumor ablation ability of V_2_C nanosheets but also provided an efficient, productive, and environmentally friendly method of disintegrating MAX, exploiting a new avenue for developing MXene with anticipated properties. Szuplewska’s group elaborated on the usage of PEG-coated Ti_2_C sheets as an emerging, effective, and selective photothermal agent, with a photothermal conversion efficiency of 87.1% under 808 nm laser irradiation [42]. It was discovered that PEG-modified Ti_2_C flakes had a negligible effect on healthy cells and tissues and may efficiently induce cancer cell ablation at a dose 24 times lower than other MXene photothermal treatments.

Apart from MXene flakes, MXene QDs have garnered considerable attention from researchers on account of their intriguing physicochemical properties, such as improved dispersion, photoluminescence, and ease of modification or doping [114]. Tetrabutylammonium hydroxide (TBAOH, (C_4_H_9_)_4_NOH) was used as an etchant by Yu’s team to create F-free Ti_3_C_2_ QDs with excellent biocompatibility and high PTT effectiveness against cancer [41]. They adopted the F-free method to modify a large number of alumina anions on the surface of MXene QDs, resulting in a strong and widespread NIR absorption. The photothermal conversion efficiency of Ti_3_C_2_ MXene QDs as prepared was found to be as high as 52.2%. Cao et al. constructed 2D V_2_C-QDs and loaded them into engineered arginine-glycine-aspartate exosomes after surface modification with cell nucleus-target TAT peptides (V_2_C-TAT) [34]. In this way, they were able to acquire the dual-targeting diagnostic and therapeutic vector V_2_C-TAT@Ex-RGD that targets the tumor cell membrane and nucleus. *In vivo* and *in vitro* studies had shown that V_2_C-TAT@Ex-RGD almost completely killed tumors under laser irradiation in the NIR-II region. This nucleus-target low-temperature PTT approach in the NIR-II region had the benefit of adequate penetration depth and few side effects.

#### 3.3.3 Synergistic photonic therapy

The variety, heterogeneity, and complexity of malignancies make single-modality therapeutic approaches frequently ineffective. Researchers have shown a great deal of interest in a synergistic treatment that combines two or more therapeutic techniques in order to increase the effectiveness of cancer treatment, decrease medication toxicity, and inhibit multidrug resistance. As an important component of phototherapy, photoactive substances are usually used for the single purpose of producing ROS as photosensitizers or generating heat as photothermal agents. MXenes have the potential to develop into multifunctional nanomaterials with photothermal effects and ROS generation properties under NIR radiation as a result of their distinctive features.

As previously described, the PEG-modified Ti_2_C with photothermal transition properties was able to selectively ablate cancer cells [42]. This phenomenon may be caused by the production of ROS in tumor cells triggered by MXenes, which exactly demonstrated the synergistic effect of PTT/PDT. Furthermore, Mo_2_C nanospheres can trigger both thermal therapy and ROS generation during NIR irradiation, which results in synergistic PTT and PDT (Figure 11E) [43]. Results from cellular experiments showed that PDT/PTT was more effective in killing tumor cells than either PDT or PTT. Furthermore, by surface modifying Ti_3_C_2_ nanosheets with DOX and HA, Liu’s team constructed a multifunctional nanoplatform that enables PDT/PTT/chemotherapy [44]. The as-prepared Ti_3_C_2_-DOX proved effective in killing tumor cells and tissues due to its stimulated responsive drug release, tumor-specific accumulation, and excellent biocompatibility (Figure 11F). Additionally, Bai’s team established Ti_3_C_2_@Met@CP, a nanoconforming drug delivery system capable of PTT/PDT/chemotherapy, by layer-by-layer adsorbing metformin (Met) and complex polysaccharides (CP) on the surface of Ti_3_C_2_ nanosheets [45]. In addition to its high photothermal conversion efficiency (59.6%) and efficient ROS producer, the CP contained in this composite nanosheet system was also a novel immunomodulator that can restrain tumor recurrence and metastasis by triggering the immune system. More recently, Ti_3_C_2_ nanosheets modified by IR780 were used to achieve synergistic treatment of laryngeal cancer with PTT and PDT, which has an excellent ability to generate ROS and disrupt mitochondrial function, according to data from both electron spin resonance and molecular biology results [46]. After 21 days of treatment, the experimental group showed an 88.1% reduction in tumor microvessel distribution and a 92% suppression of tumor growth compared to the control group.

### 3.4 Photonic theranostics

Theranostics is thought to be an efficient way to provide real-time disease surveillance and diagnosis since it can merge diagnostic imaging and therapy modalities [115–117]. 2D MXenes nanosheets feature a considerable deal of potential for use in the treatment of diseases due to their distinctive physicochemical characteristics, ultra-thin nanostructures, and manageable chemical components. The therapeutic effect of 2D MXenes can be enhanced by further surface modification of diverse molecules or nanomaterials, expanding their biological applications. Over the years, great efforts have been made to explore the application of 2D MXenes nanosheets in cancer therapy. Up to now, the typical 2D MXenes nanosheets, including Ti_3_C_2_, Ta_4_C_3,_ Mo_2_C, and V_2_C, have been reported to be effective for cancer therapy.

**Figure 12:**
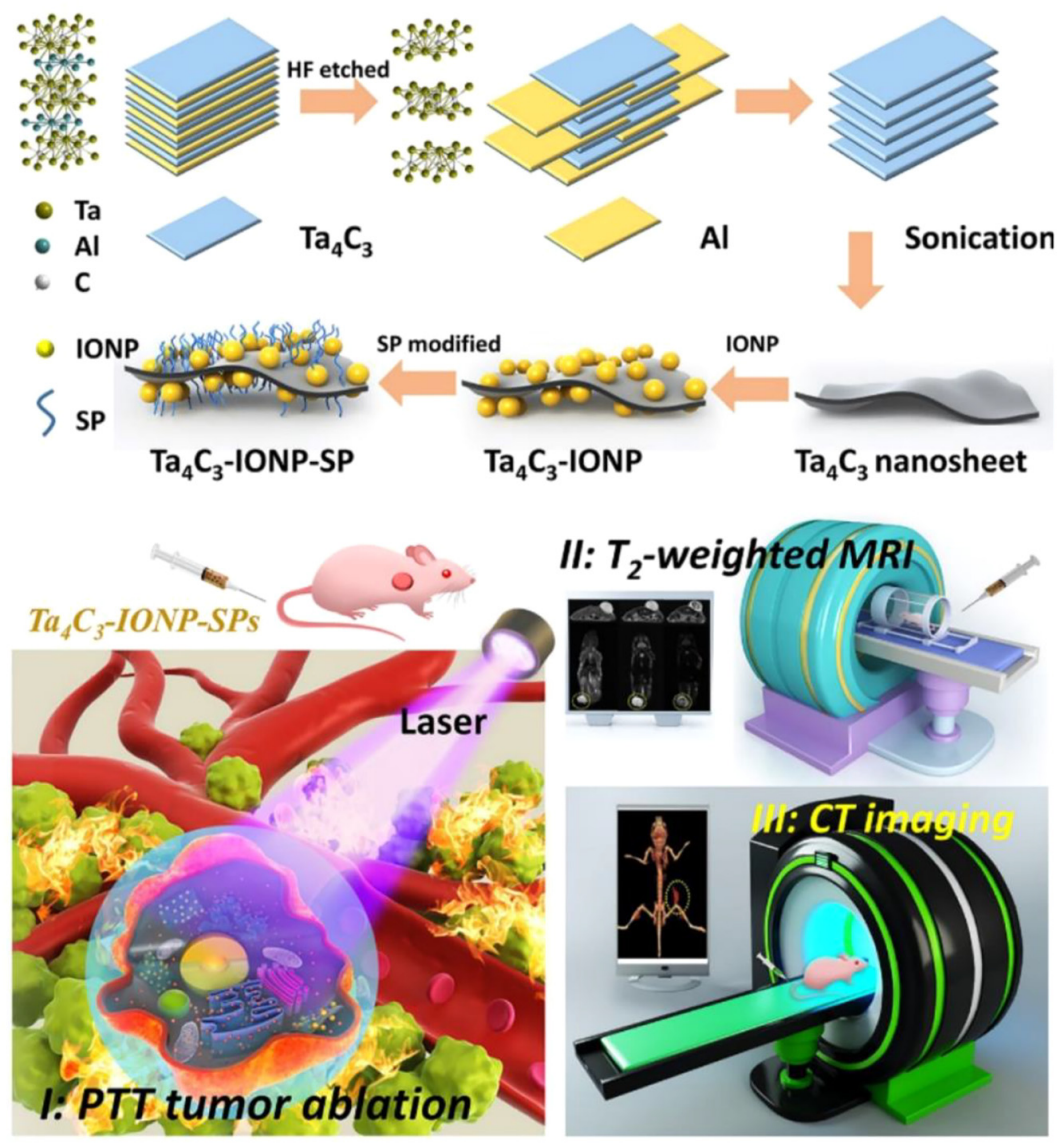
Schematic representation of the construction of Ta_4_C_3_-IONP-SPs nanosheet and MRI/CT dual-modality imaging-guided tumor therapy [36]. Copyright 2018, Wiley-VCH.

As a representative 2D MXene, Ti_3_C_2_ and its composite nanomaterials have been investigated for cancer therapy. As described earlier, the Ti_3_C_2_@Au nanocomposites modified by Au nanoparticles and PEG can achieve PA/CT dual-mode imaging-guided PTT in combination with radiotherapy without evident long-term toxicity at an administered dose [47]. Moreover, Fe(II)-Ti_3_C_2_ nanoshells synthesized by appropriate Fe^2+^ doping efficiently achieved MRI imaging-guided PTT/chemodynamic therapy against MKN45 tumor [48]. With regard to Ta_4_C_3_, Ta_4_C_3_-IONP-SP composite nanosheets that enable MR imaging are capable of PTT of breast cancer, while MnO_*x*_/Ta_4_C_3_ composite nanosheets are featured with MR/CT/PA tri-modality imaging capacity [24, 36] (Figure 12). Beyond Ti_3_C_2_ and Ta_4_C_3_ MXenes, V_2_C and its complex, V_2_C-TAT@Ex-RGD, have also been proved to be effective against MCF-7 breast cancer [34]. *In vitro* and *in vivo* experiments demonstrated that high-quality V_2_C nanosheets fabricated through a green layering approach were able to achieve efficient tumor suppression with the guidance of dual-mode imaging by PA and MRI [49]. V_2_C-TAT@Ex-RGD with fluorescence/PA/MRI capability exhibited low-temperature PTT [34]. Notably, V_2_C-TAT@Ex-RGD nanosheets with high biocompatibility and prolonged circulation time can target the nucleus of cancer cells with high transfection effect and desirable intranuclear body escape ability.

## 4 Current challenges and new opportunities

**Figure 13:**
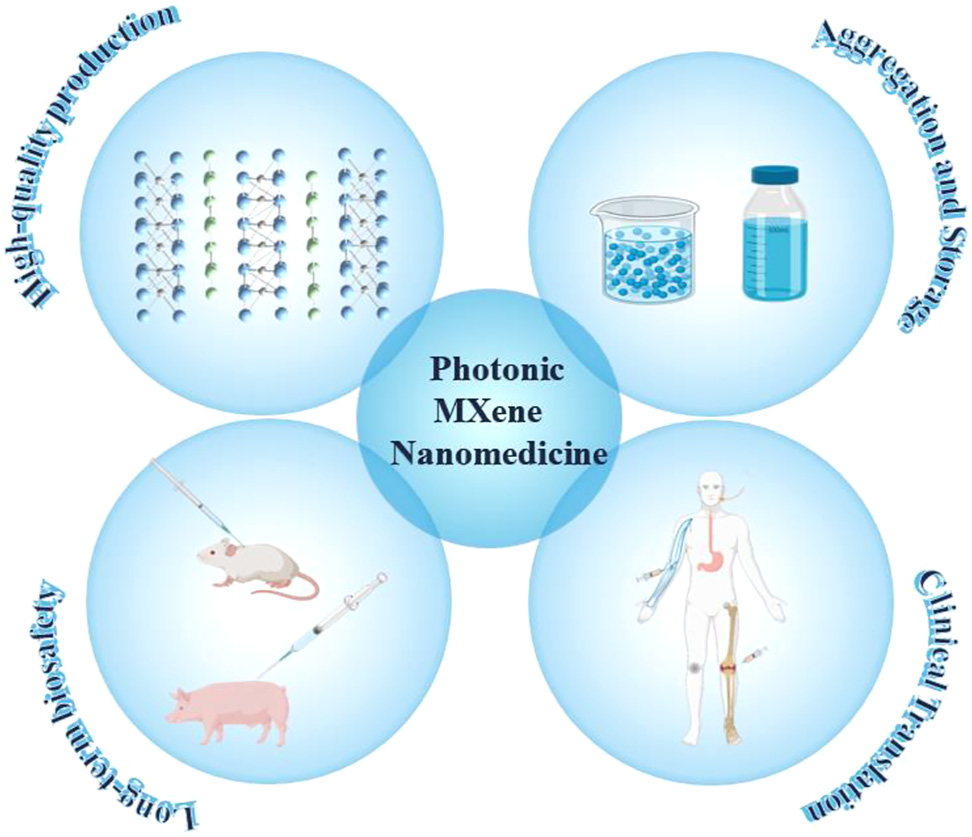
Schematic diagram of the developments and challenges of 2D MXenes for biomedical applications, including mass production, aggregation and storage, long-term biosafety, and clinical translation.

2D MXenes have received a lot of attentions from researchers due to their large surface area, diverse functionalization, desirable optical property, and excellent biocompatibility since their discovery in 2011. It is worth mentioning that the development of 2D MXene nanomaterials for nanomedicine is still in its infancy. This review summarizes and discusses the current progress on the preparation and functionalization of MXenes and their composite counterparts, with an emphasis on its photo-biomedical applications, including photonic antibacteria, photonic bio-imaging, photonic therapy, and theranostics. In terms of the preparation process, the available 2D MXene nanosheets were primarily made from etching MAX phase precursors, which were subsequently exfoliated to create MXene layers with adjustable physicochemical and nanostructural features. In general, varying functionalities of MXenes are determined by different compositions, sizes, and/or surface status. For example, high atomic number metals such as Ta and W allow MXenes to feature superior X-ray/CT contrast capabilities, thus enabling CT imaging, while MXenes with transition metals such as Ti, Ta, and Nb as M typically exhibit excellent biocompatibility. In addition, MXene modified by surface modification with PEG, PVP, folic acid, and hyaluronic acid have different hydrophilic and targeting abilities, while MXene modified by surface functionalization with nanoparticles such as Ag, Au, MnO_*x*_, and triiron tetroxide can be applied to therapeutic and diagnostic imaging of diseases. Despite the emerging 2D MXene nanosheets exhibit excellent structural, physiochemical, and theranostic properties, such as high hydrophilicity, desirable biocompatibility, large surface area-to-volume ratio, and high photothermal conversion efficiency, there are still several challenges and critical issues those should be elucidated and resolved for further clinical translation (Figure 13).

(1) Although both top-down and bottom-up synthesis methods have been used to prepare MXene materials, bottom-up remains the dominant method. However, it is difficult to precisely control the composition and structure of 2D MXene nanosheets prepared through this technique. Moreover, the as-prepared MXene nanosheets are not stable in physiological condition. Appropriate surface modifications are necessary to effectively develop the diverse functions and stability of MXenes. Especially, the high-quality and large-scale production of MXene remains a challenge to address.
(2) Long-term biocompatibility and biosafety are critical for the utilization of MXene in biomedical applications. In the available studies, the vast majority of MXene exhibited mild toxicity and a small percentage exhibited biodegradability. It is worth mentioning that most of the studies have been performed *in vitro* or on tiny animals for a brief period of time using low-dose MXene materials. Therefore, long-term systematic biosafety evaluation (*e.g.*, biocompatibility, pharmacokinetics, biodistribution, immunity, degradability, etc.) in large animals is necessary to achieve a broader range of biological applications and reveal the underlying biological effects/biosafety.
(3) MXenes and MXene-based compounds may precipitate or agglomerate in biological media due to their instability. The utilization of surface-modified organic polymers (like PVP and SP) to enhance the stability and dispersion of MXene in various physiological settings should thus be investigated. In addition, more researches must be done on the inventive design of multifunctional nanostructures based on MXene that have higher stability and selectivity/sensitivity.
(4) Compared with other 2D materials, MXenes exhibited a unique surface structure, controlled composition, tunable optical, electrical, and magnetic properties. However, up to now, the working mechanism of MXene-mediated therapy is not fully understood. It is necessary to comprehensively understand the corresponding physiochemical properties and biological effects of MXenes to enhance their potential for clinical translation and biomedical applications.

Therefore, despite it seems that MXenes are currently demonstrating with great potential for biomedical applications on disease treatments, there are still a lot of obstacles to clear. And it is still a challenging procedure to move MXenes from fundamental research to clinical utilization. On this ground, significant efforts should be devoted to the rational engineering of 2D MXene-based nanomedicine and biomaterials with optimized and desirable theranostic performance for satisfying the biomedical requirements in clinic.
